# Micropeptide YG-6 encoded by exosomal LINC01123 derived from highly migratory ovarian cancer cells promotes tumor progression

**DOI:** 10.1186/s12943-026-02621-w

**Published:** 2026-03-05

**Authors:** Han Lei, Zhengwei Zhou, Chengyuan Li, Lili Fan, Qihan Wu, Maonan Wang, Ke Guo, Qiong Pan, Gil Mor, Guang Shu, Juanni Li, Gang Yin

**Affiliations:** 1 Department of Pathology, Xiangya Hospital, Xiangya School of Basic Medical Sciences, Central South University, Changsha, Hunan China; 2 Department of Obstetrics and Gynecology, The Third Xiangya Hospital of Central South University, Changsha, Hunan China; 3 Department of Neurology, The Third Xiangya Hospital of Central South University, Changsha, Hunan China; 4 Guangzhou Key Laboratory of Formula-Pattern of Traditional Chinese Medicine, School of Traditional Chinese Medicine, Jinan University, Guangzhou, Guangdong China; 5 Shanghai-MOST Key Laboratory of Health and Disease Genomics, NHC Key Lab of Reproduction Regulation, Shanghai Institute for Biomedical and Pharmaceutical Technologies, Shanghai, China; 6 C.S. Mott Center for Human Growth and Development, Wayne State University, 275 E Hancock Av, Detroit, MI 48201 USA; 7 China-Africa Research Center of Infectious Diseases, Xiangya School of Basic Medical Sciences, Central South University, Changsha, Hunan China; 8 National Clinical Research Center for Geriatric Disease (Xiangya Hospital), Central South University, Changsha, Hunan China

**Keywords:** Ovarian Cancer, Exosomal LINC01123, Micropeptide, YG-6, Migration, Focal adhesion

## Abstract

**Background:**

Intercellular crosstalk plays a pivotal role in tumor progression and metastasis. Exosomes can package long non-coding RNAs (lncRNAs) to mediate extracellular communication. Although exosome research in various cancers has made remarkable progress, it remains unclear in ovarian cancer (OC) whether the cellular communication between OC cells with different metastatic potentials influences progression via exosomal-lncRNA. Besides, lncRNA can encode functional micropeptides, yet the association between OC and micropeptides encoded by exosomal-derived lncRNA has not been reported.

**Methods:**

OC cell-derived exosomes were characterized using transmission electron microscopy, nanoparticle tracking analysis, and WB. Exosomal lncRNA sequencing results of highly migratory OC cells (HMOs) and low migratory OC cells (LMOs) were analyzed, and LINC01123 was selected for further study. The effect of exosome LINC01123 on the function of LMOs was detected by the co-culture experiment. Bioinformatics prediction, mass spectrometry (MS), WB, and a series of functional experiments showed that LINC01123 encodes a small 59 aa peptide, named YG-6 (LINC01123 Yield peptide Gaining metastasis function: ORF no.6). Immunofluorescence, qPCR, WB, immunohistochemistry, and MS analysis were used to detect the endogenous expression of YG-6 in OC cells and tissues. The function of YG-6 in OC was investigated by detecting cell migration ability and observing tumor metastasis in nude mice. Mechanistically, the relationship between the YG-6 and ACTC1 in OC was verified by Co-Immunoprecipitation, WB, MS, immunofluorescence, and functional experiment.

**Results:**

LINC01123 was significantly up-regulated in exosomes derived from HMOs and was readily internalized by LMOs, promoting their malignant behavior. Interestingly, LINC01123 encodes a small peptide composed of 59 amino acids, named YG-6, which exhibits endogenous high expression in OC cells and tissues. Functional experiments demonstrated that LINC01123 promotes tumor progression by encoding the peptide YG-6, rather than via an RNA-dependent mechanism. Furthermore, we identified ACTC1 as a binding protein of YG-6, which can synergistically activate the focal adhesion signaling pathway together with YG-6, thereby promoting the migration and adhesion of LMOs.

**Conclusions:**

Our findings not only demonstrate the YG-6 peptide encoded by LINC01123 as a potential prognostic biomarker for OC but also uncover a new mechanism of OC progression driven by YG-6 peptides.

**Supplementary Information:**

The online version contains supplementary material available at 10.1186/s12943-026-02621-w.

## Introduction

Metastasis and recurrence, responsible for approximately 90% of cancer-related deaths, are hallmark features of malignant tumors [1–3]. In recent years, the global incidence of ovarian cancer (OC) has continued to rise, contributing to high mortality rates and significantly affecting women’s health worldwide [4–6]. Most OC cases present with no distinct clinical symptoms in the early stages, leading to diagnosis at more advanced stages characterized by disseminated intra-abdominal metastasis. Compared with the primary tumor itself, metastatic disease is more fatal [7, 8]. Accordingly, identifying key metastasis-driving genes and understanding their underlying mechanisms, as well as discovering effective therapeutic targets to prevent metastasis, is crucial for developing treatment strategies that improve the prognosis of OC patients.

Intercellular communication plays a crucial role in cancer progression and metastasis. Exosomes (Exos), which serve as effective mediators of intercellular signaling, have emerged as significant promoters of cancer invasiveness in recent years [9, 10]. These nanovesicles, characterized by lipid bilayer membranes, contain a diverse range of contents, including proteins, lipids, and nucleic acids. They are derived from multivesicular bodies and can be readily purified from culture media and biological fluids, such as serum, ascites, and urine [11, 12]. Studies have shown that exosomes from highly metastatic melanoma cells can enhance the malignancy of low-metastatic melanoma cells by cultivating bone marrow progenitor cells [13]. Another study also reported that exosomes containing S100A4 from highly metastatic hepatocellular carcinoma cells facilitate the metastasis of liver cancer by activating STAT3 [14]. However, the role of intercellular communication via exosomes between OC cells with different metastatic potentials remains poorly understood.

Increasing evidence has revealed that exosomes derived from cancer cells play a key role in tumor cell-to-cell communication by transferring and exchanging oncogenic molecules, including proteins, lipids, miRNA, mRNA, and lncRNA [15–17]. LncRNA is a class of RNA molecules with a transcription length exceeding 200 nt [18]. Numerous studies have implicated lncRNAs in various tumor processes, including tumor proliferation, metastasis, angiogenesis, and drug resistance [19–24]. Recent advancements in the study of ncRNAs, particularly lncRNAs, have led to several breakthroughs. Traditionally, ncRNAs, which lack an open reading frame (ORF), were thought to function primarily as RNA molecules. However, several independent research studies have demonstrated that lncRNAs can encode and translate functional polypeptides or protein molecules, contributing to fundamental developmental processes across multiple organisms and regulating the onset and progression of certain diseases [25–28]. Despite these insights, the functions and mechanisms of lncRNAs remain largely unexplored. Notably, to date, there have been no research reports on the regulation of OC by micropeptides encoded by exosome-derived lncRNAs. Very little is known about how peptides activate signaling pathways that play a crucial role in diseases such as cancer, more extensive investigations are necessary to identify new micropeptides and characterize their functions and regulatory networks.

In this study, we investigated the lncRNA-Seq profile of exosomes derived from OC cells with diverse metastatic potential. Exosomal LINC01123 derived from highly migratory OC cells (HMOs) enhanced the migration abilities of low-migratory OC cells (LMOs), indicating that exosomes could mediate interplay within the tumor ecosystem, affecting phenotypical intratumor heterogeneity. Meanwhile, we found for the first time that the exosomal LINC01123 derived from HMOs encodes a conserved 59-amino acid (aa) protein. We named this protein YG-6 (LINC01123 **Y**ield peptide **G**aining metastasis function: open reading frame no.**6**) since it was found to promote the progression of OC by modulating cell migration. And it has been observed that YG-6 is endogenously highly expressed in both OC cells and tissue samples. Furthermore, we determined that the YG-6 peptide binds to ACTC1 to activate the focal adhesion signaling pathway, thereby promoting the migration and adhesion of LMOs. Collectively, our findings reveal a novel mechanism by which oncopeptide YG-6 promotes OC progression.

## Materials and methods

For a comprehensive description of the methodologies used in this study, please refer to the Supplementary Materials.

### Tissue and plasma samples

Plasma samples were obtained from 5 healthy volunteers and 8 OC patients, as well as tumor tissues along with non-tumoral tissues of OC patients who underwent tumor resection in the Department of Gynecological Oncology, Xiangya Hospital, Central South University. All patients were pathologically confirmed to have OC. Initially, blood was centrifuged at 3,000 × g for 10 min at 4 °C. The supernatant was then carefully collected and subjected to a second centrifugation (12,000 × g for 15 min at 4 °C ) to remove remaining cell debris. All plasma and tissue samples were snap-frozen in liquid nitrogen and subsequently stored at -80 °C until further analysis. Informed consent was obtained from all participants before sample collection. All experiments strictly adhered to the code of ethics of the World Medical Association and were conducted following the guidelines of Central South University.

### Anti-YG-6 antibody production

The rabbit polyclonal antibodies against the YG-6 micropeptide (59 aa) were generated by GL Biochem (Shanghai). Specifically, the epitope peptide SSEEAGSTVWEGRRC was synthesized and conjugated to Keyhole Limpet Hemocyanin (KLH) to enhance immunogenicity. Four New Zealand White rabbits were subjected to an 8-week immunization regimen: the primary immunization was performed on day 0 with 500 µL of antigen emulsified in Complete Freund’s Adjuvant (CFA), followed by seven successive boosters on days 14, 28, 35, 42, 49, 56, and 63 using CFA or Incomplete Freund’s Adjuvant (IFA) to maintain high antibody titers. Antiserum was collected on day 70 and subjected to antigen-affinity chromatography. Briefly, the antiserum was loaded onto a column pre-equilibrated with Tris-EDTA buffer; after washing, the bound antibodies were eluted with glycine buffer, neutralized, and dialyzed in 1× PBS buffer at 4 °C for 48 h. The final concentration of the purified anti-YG-6 antibody reached approximately 0.40–0.49 mg/mL, with a verified ELISA titer exceeding 1: 64,000, ensuring high specificity and affinity for downstream applications.

### Western blotting analysis

Whole-cell lysates and tissue lysates were prepared, and the protein concentrations were determined as previously described [29]. For standard protein analysis, lysates were separated using 10% Tris-Glycine SDS-PAGE. The low molecular weight peptide was separated on 16% Tricine-SDS-PAGE as previously described [30]. For the high-resolution separation of low molecular weight peptides (2.5–40 kDa), a specialized Tricine-SDS-PAGE system (Coolaber, SK6012) was employed. Specifically, a 16% resolving gel (16% T, 6% C) and a 4% stacking gel (4% T, 3% C) were prepared using the provided acrylamide/bisacrylamide solutions and 3× gel buffer. Given that the target molecular weight was approximately 10 kDa, a two-layer gel configuration was utilized as it provides sufficient resolution for proteins greater than 5 kDa. Protein samples were mixed 1:1 with 2× Tricine peptide loading buffer, denatured at 100 °C for 8 min. Electrophoresis was conducted in a dual-buffer system, using 1× cathode buffer (containing Tricine and SDS) for the inner chamber and 1× anode buffer (0.2 M Tris) for the outer chamber. For precise molecular weight determination, a pre-stained low-molecular-weight protein marker (Jingxin Biology, China, Cat: JX180401) was loaded alongside the samples. The separation was carried out at a constant voltage of 30 V for 1 h, followed by 150 V for 4 h. Subsequently, the peptides were electroblotted onto PVDF membranes at a constant current of 200 mA for 40–50 min in a chilled transfer buffer. Following the transfer, the membranes were blocked with 5% bovine serum albumin (BSA) in 1× TBST for 2 h at room temperature to minimize non-specific binding. The membranes were then incubated with specific primary antibodies overnight at 4 °C, followed by secondary antibody incubation and chemiluminescence detection.

The indicated proteins were detected using anti-YG-6 (our developed, 1:500), Flag (Abcam, ab205606, 1:2000), Flag (Utibody, UM3009, 1:2000), GFP (Utibody, UM3003, 1:2000), ACTC1 (Immunoway, YM8082, 1:1000), ACTC1 (Cloud-Clone, CAB341Hu22, 1:1000), FAK (Immunoway, YM8154, 1:1000), p-FAK (Immunoway, YM8684, 1:1000), Paxillin (Immunoway, YM8177, 1:1000), p-Paxillin (Immunoway, YM8268, 1:1000), SRC (HUABIO, ET1702-03, 1:1000), p-SRC (Immunoway, YM8471, 1:1000), Vimentin (HUABIO, ET1610-39, 1:2000), and GAPDH (UM4002, Utibody, 1:2000) antibodies. For quantitative western blotting (WB) analysis, all experiments were independently performed in triplicate. Gray value analysis of the protein bands was conducted using ImageJ software, with the target protein levels were normalized to the corresponding loading controls. Subsequently, the relative protein abundance was calculated as a fold-change relative to the control group for statistical evaluation.

### Animal care and ethics statement

5-week-old female nude mice were purchased from the Experimental Animal Center of Central South University (Changsha, Hunan, China). An in vivo abdominal transfer experiment was performed using previously described methods [29]. Briefly, nude mice in each experimental group were injected with 1 × 10^7^ HO-8910-LV-YG-6, HO-8910-LV-5U-YG-6, and HO-8910-LV-5U-YG-6mut cells through the intraperitoneal injection (*n* = 5). Nude mice were euthanized at the end of the experiment (30 days after cell injection), the metastatic nodules were counted, and the RNA and protein of the mice’s tumor tissue were extracted to detect the corresponding indexes. All experimental protocols concerning the handling of mice were approved by the institutional animal care and use committee of Central South University. The nude mice used in this study were bred and maintained under defined conditions at the Animal Experiment Center (SPF grade), Central South University. Mice were housed in a temperature-controlled (22 °C) and light-controlled pathogen-free animal facility with free access to food and water.

### Statistical analysis

All experiments were repeated in triplicate unless stated in the figure legend. Quantitative results of statistical analysis are expressed as the mean ± standard deviation (SD) of at least three independent experiments. All statistical analyses were performed using GraphPad Prism 10.1.2 software (GraphPad Software, USA). The statistical significance of differences between two independent groups was evaluated using an unpaired Student’s t-test, whereas comparisons among three or more experimental groups were analyzed by one-way analysis of variance (ANOVA) followed by Dunnett’s or Tukey’s post-hoc test. Survival curves were analyzed with the log-rank test using the Kaplan-Meier method. Significance levels were defined as follows: * for *p* < 0.05, ** for *p* < 0.01, *** for *p* < 0.001, **** for *p* < 0.0001. A *p*-value of less than 0.05 was considered to indicate a statistically significant difference, while “ns” represents no statistical significance.

## Results

### Highly migratory OC cells (HMOs) derived exosomes enhance the metastatic potential of low migratory OC cells (LMOs)

**Fig. 1:**
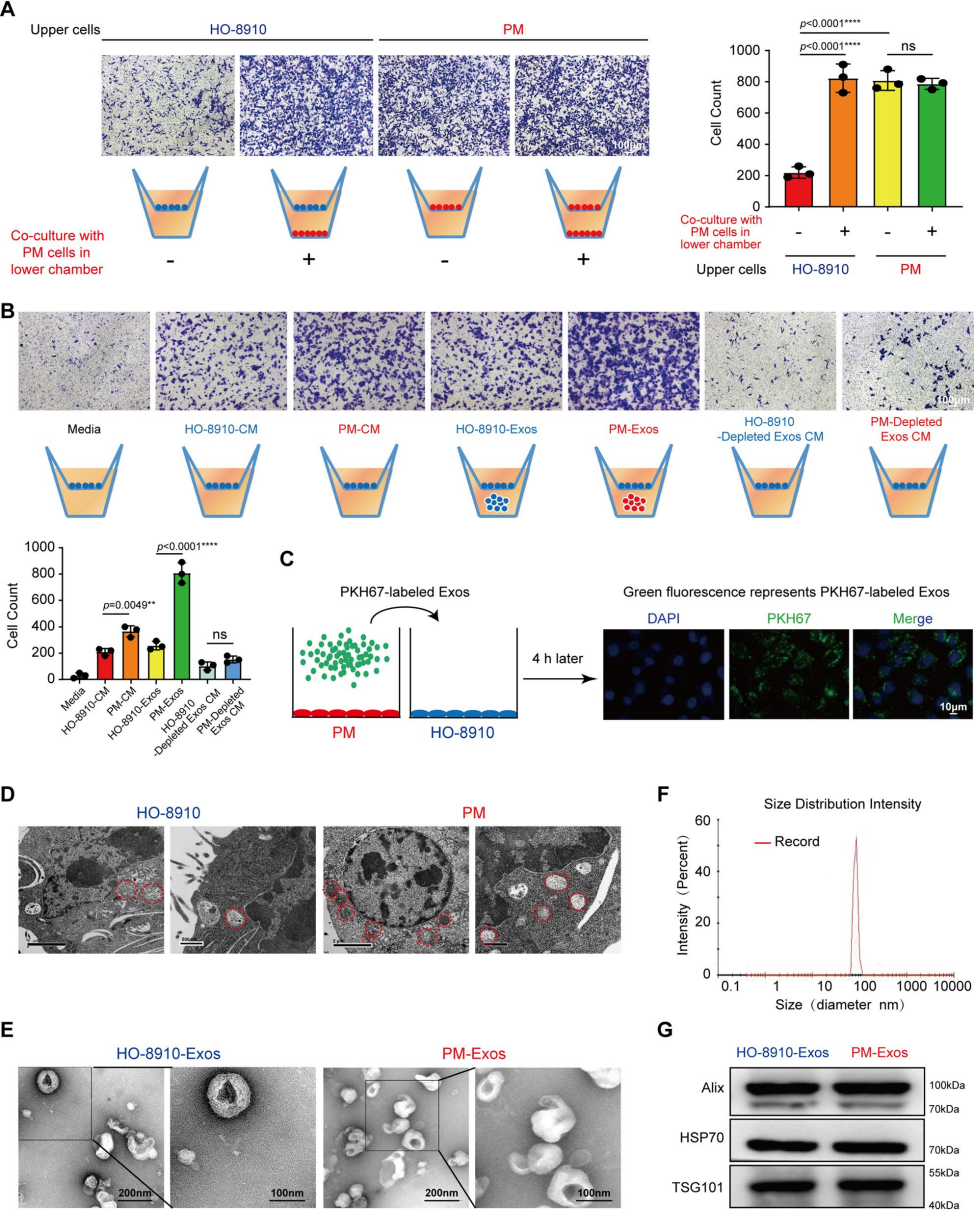
HMOs-derived exosomes enhance the metastatic potential of LMOs. **A** Representative images of cell migration in the co-culture group treated with HO-8910 or PM. HO-8910 cells in blue, PM cells in red. Scale bar: 100 μm (Left). The number of cells was counted by ImageJ (Right). **B** Transwell assays detected HO-8910 cell migration in the co-culture group treated with Media (1640), HO-8910-CM, PM-CM, HO-8910-Exos (50 µg/mL), PM-Exos (50 µg/mL), HO-8910-Depleted Exos CM, and PM-Depleted Exos CM. Scale bar: 100 μm. The number of cells was counted by ImageJ. **C** The exosomes were isolated from the medium of PM. PKH67-labeled PM-Exos can be endocytosed into the recipient cells HO-8910, and the nuclear DNA was stained with DAPI. Scale bar: 10 μm. **D** Electron microscopy images show representative fields with MVBs (Red circles) in HO-8910 and PM, respectively. Scale bar: 2 μm (Left) and 500 nm (Right). **E** Representative TEM images of purified exosomes from HO-8910 and PM. Scale bar: 200 nm (Left) and 100 nm (Right). **F** NanoSight particle tracking analysis of the size distributions and number of exosomes. **G** Western blotting (WB) detected Alix, HSP70, and TSG101 in exosomes secreted from PM and HO-8910. The data are represented as the mean ± SD from three independent experiments (**A**,** B**), and the *p* value was determined by one-way ANOVA followed by Tukey’s post-hoc test (**A, B**). Each experiment was performed in triplicate. **p < 0.01 or ****p < 0.0001, ns indicates no significance

Cancers, including ovarian cancer (OC), frequently display substantial intra-tumor heterogeneity in virtually all distinguishable phenotypic features. Beyond this heterogeneity, exosomes (Exos) have emerged as key players in facilitating cancer metastasis and invasiveness in recent years [16]. To determine cell-to-cell communication, we established an in vitro model consisting of two OC cell lines with differential migratory capacity and two migration chamber wells. Subsequently, we selected the HO-8910PM cells (short name PM) with stronger migration ability and the HO-8910 cells with relatively weaker migration ability (Fig. S1). First, the highly migratory OC cells (HMOs) PM were plated in the lower chamber, while the low migratory OC cells (LMOs) HO-8910 were plated in the upper chamber. After 24 h, the presence of migratory cells was quantified by cell imaging. Interestingly, we observed that after co-culturing HO-8910 with PM, it exhibited stronger migration capabilities compared to the control group (Fig. 1A). These data suggest that signals originating from PM cells can direct the low migratory HO-8910 cells.

Next, we tested whether the secretions from PM cells are responsible for the observed HO-8910 migration. To test this hypothesis, we used the same two-chamber models with the low migration HO-8910 cells plated in the upper part of the chamber and conditioned medium (CM) obtained from PM cells. After 24 h, the transwell migration assay revealed that PM-CM significantly enhanced the migratory capacity of receptor HO-8910 cells compared to either regular media or CM derived from HO-8910 cells themselves (Fig. 1B). We hypothesize that exosomes could be the major communication factors secreted by HMOs with the capacity to enhance cell migration. To test this hypothesis, we isolated exosomes from HO-8910 and PM cells by high-speed ultracentrifugation (Figs. S2A-B) and used the two-cell chamber where HO-8910 cells were plated in the upper chamber and the exosomes in the lower chamber for 24 h. We observed a significant increase in the number of HO-8910 migrating cells in the chambers containing exosomes from PM cells (Fig. 1B). The depleted-Exos CM from PM cells did not affect HO-8910 cells’ migration (Fig. 1B). These data indicate that factors present in the exosomes from PM cells are responsible for the induction of cell migration.

To further confirm that the exosomes can reach and be internalized by HO-8910 cells, we labeled PM-derived exosomes with the exosome green fluorescent labeling dye PKH67 and co-cultured them with HO-8910 cells for 4 h. Using fluorescence microscopy, we observed positive fluorescence signals in HO-8910 cells that had been exposed to PM-derived exosomes labeled with PKH67 (Fig. 1C). Then, to identify the presence of exosomes in PM and HO-8910 cells, we used transmission electron microscopy (TEM) and found multivesicular bodies (MVBs) in both cells, although, the number of MVBs seems to be higher in PM cells (Fig. 1D). Finally, we confirm the structural features of purified exosomes by TEM and NanoSight analysis (NTA), we observed the presence of 30–150 nm exosomes in preparations from PM and HO-8910 cells (Figs. 1E-F). The purity of the exosome preparation was confirmed by the expression of exosome-specific protein markers Alix, Hsp70, and TSG101. All these markers were detected in the exosomes isolated from HO-8910 and PM cells (Fig. 1G). These results suggested that the pro-migratory effect of PM-Exos was mediated by their specific bioactive cargos rather than the exosomal structure.

### Identification of LINC01123 as a potential factor inducing cell migration

**Fig. 2:**
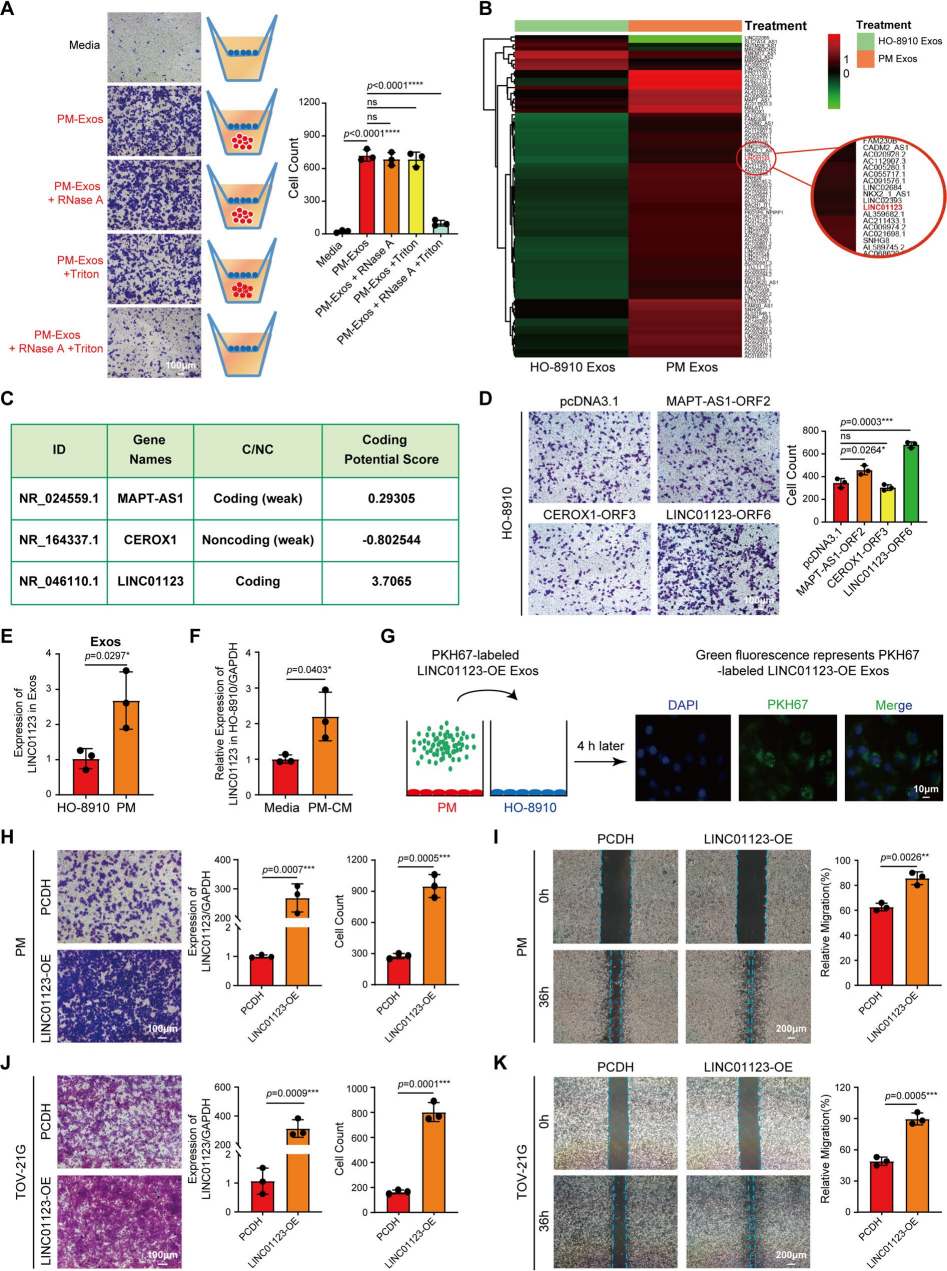
Identification of LINC01123 as the potential factor inducing cell migration. **A** Representative images of cell migration in the co-culture group treated with Media, PM-Exos, or PM-Exos+RNase A, PM-Exos+Triton, and PM-Exos+RNase A+Triton. HO-8910 cells in blue. Scale bar: 100 μm (Left). PM-Exos (50 µg/mL) were either treated with RNase A (20 µg/mL) at 37 °C for 30 min to degrade extra-exosomal RNA, or pre-incubated with 0.1% Triton X-100 at room temperature for 15 min to disrupt the exosomal membranes prior to RNase A digestion. The bar graph presents the quantitative analysis of migrated cells performed using ImageJ software (Right). **B** Exos-RNA-Seq (|log_2_FC| > 5, *p* < 0.05) sequencing analysis compared the different lncRNAs extracted from HO-8910-Exos and PM-Exos. **C** The CPC online website predicts the coding capability scores of MAPT-AS1, CEROX1, and LINC01123. **D** Detection of migration ability of 3 candidate lncRNA-ORF with coding potential in OC cells. Scale bar: 100 μm. **E** qPCR was used to detect LINC01123 expression in HO-8910-Exos and PM-Exos. **F** The expression of LINC01123 in HO-8910 was detected by qPCR after 36 h incubation of PM-CM with HO-8910. **G** The exosomes are isolated from the medium of PMLINC01123−OE stable cells (LINC01123-OE Exos). LINC01123-OE Exos were labeled with PKH67 and the nuclear DNA was stained with DAPI. HO-8910 cells were incubated with LINC01123-OE Exos for 4 h. Scale bar: 10 μm. **H**-**K** Transwell assay (**H**, **J**) and wound healing assay (**I**, **K**) were used to assess the impact of LINC01123-OE on OC cell migration abilities. Scale bar: 100 μm (**H**, **J**) and 200 μm (**I**, **K**). The data are represented as the mean ± SD from three independent experiments (**A**, **D**-**F**, **H**-**K**), and the *p* value was determined by two-tailed Student’s t test (**E**-**F**, **H**-**K**) or one-way analysis of variance (ANOVA) followed by Tukey’s (**A**) or Dunnett’s post-hoc test (**D**). Each experiment was performed in triplicate. *p < 0.05, **p < 0.01, ***p < 0.001, or ****p < 0.0001, ns indicates no significance

Exosome content includes proteins, lipids, microRNA, mRNA, and lncRNAs [31]. We sought to determine the exosome cargos responsible for the induction of cell migration. Thus, we isolated exosomes from PM cells and exposed it to HO-8910 cells using the two-chamber migration assay, PM-Exos increased significantly HO-8910 cell migration (Fig. 2A). Moreover, the induction of HO-8910 cell migration by PM-Exos was not significantly altered by treatment with RNase A alone, indicating that the functional RNA molecules are protected from external degradation by the intact exosomal lipid bilayer (Fig. 2A). However, when PM-Exos were pre-treated with Triton X-100 to disrupt the membrane structure, subsequent exposure to RNase A abolished their ability to stimulate cell migration (Fig. 2A). These data suggest the existence of specific RNA present in the PM-Exos responsible for the induction of cell migration. To identify the specific RNAs mediating the migratory effect, we performed RNA-Seq in exosomes isolated from HO-8910 and PM cells. We found a significant difference in the RNA content between the two types of exosomes (Fig. 2B). Interestingly, we found a high number of lncRNAs differentially expressed between the two types of exosomes. A total of 83 differentially expressed lncRNAs were screened with |log_2_FC| > 5 (Fig. 2B). Increasing evidence suggests that short open reading frames (ORFs) in lncRNAs can encode functional peptides [32]. To identify lncRNAs with encoding potential in OC, we screened out a total of 9 lncRNAs with potential coding ability from 83 lncRNAs through bioinformatics (Fig. S3A). Next, we conducted further screening through additional criteria, and the screening condition was that (1) High expression in OC cells; (2) Expression was higher in PM compared to HO-8910. Therefore, we focused on the characterization of the top 3 lncRNAs with potential coding ability: MAPT-AS1, CEROX1, and LINC01123. The nucleotide sequences and amino acid sequences of the putative ORFs within these lncRNAs are documented in Table S3. To further quantify their coding capacity, we utilized the Coding Potential Calculator (CPC) online platform (https://cpc.gao-lab.org/). Among the candidates, LINC01123 yielded the highest coding potential score, providing that this lncRNA may encode a novel and functional regulatory peptide (Fig. 2C).

We posit that some of these lncRNAs may encode peptides that then induce the migration. To test this hypothesis, we clone the ORF of each of these lncRNAs into the pcDNA3.1 plasmid. The constructs were then transfected into HO-8910 cells, and the migratory capacity was determined using the two-chamber assay. As shown in Figs. 2D and S3B, transfection with LINC01123-ORF6 induces the significant migration of HO-8910 cells compared to the control empty plasmid. A minimal effect was observed with other constructs (Figs. 2D, S3B). Next, we validated the presence of LINC01123 in HO-8910 and PM-derived exosomes by qPCR, and observed, as expected, high levels of LINC01123 on PM-derived exosomes (Fig. 2E). Afterward, we exposed HO-8910 cells to PM-CM and determined the expression levels of LINC01123 by qPCR. We found higher levels of LINC01123 expression on HO-8910 cells incubated with PM-CM, further confirming the transfer of exosomes containing LINC01123 into HO-8910 cells (Fig. 2F). To further confirm the transfer of LINC01123, we overexpressed LINC01123 in PM cells, isolated exosomes and labeled them with PKH67. Then, HO-8910 cells were exposed to PKH67-labeled LINC01123 overexpressing exosomes, and fluorescence was monitored by microscopy. Four hours after inculcation, we can already observe the presence of PKH67-positive cells, further confirming the transfer of LINC01123 containing exosomes (Fig. 2G). Besides, functional experiments also showed that overexpression of LINC01123 could significantly promote the migration of OC cells (Figs. 2H-K) while silencing of LINC01123 suppresses OC cell migration (Fig. S3C).

### Differential expression of LINC01123 in OC patients

**Fig. 3:**
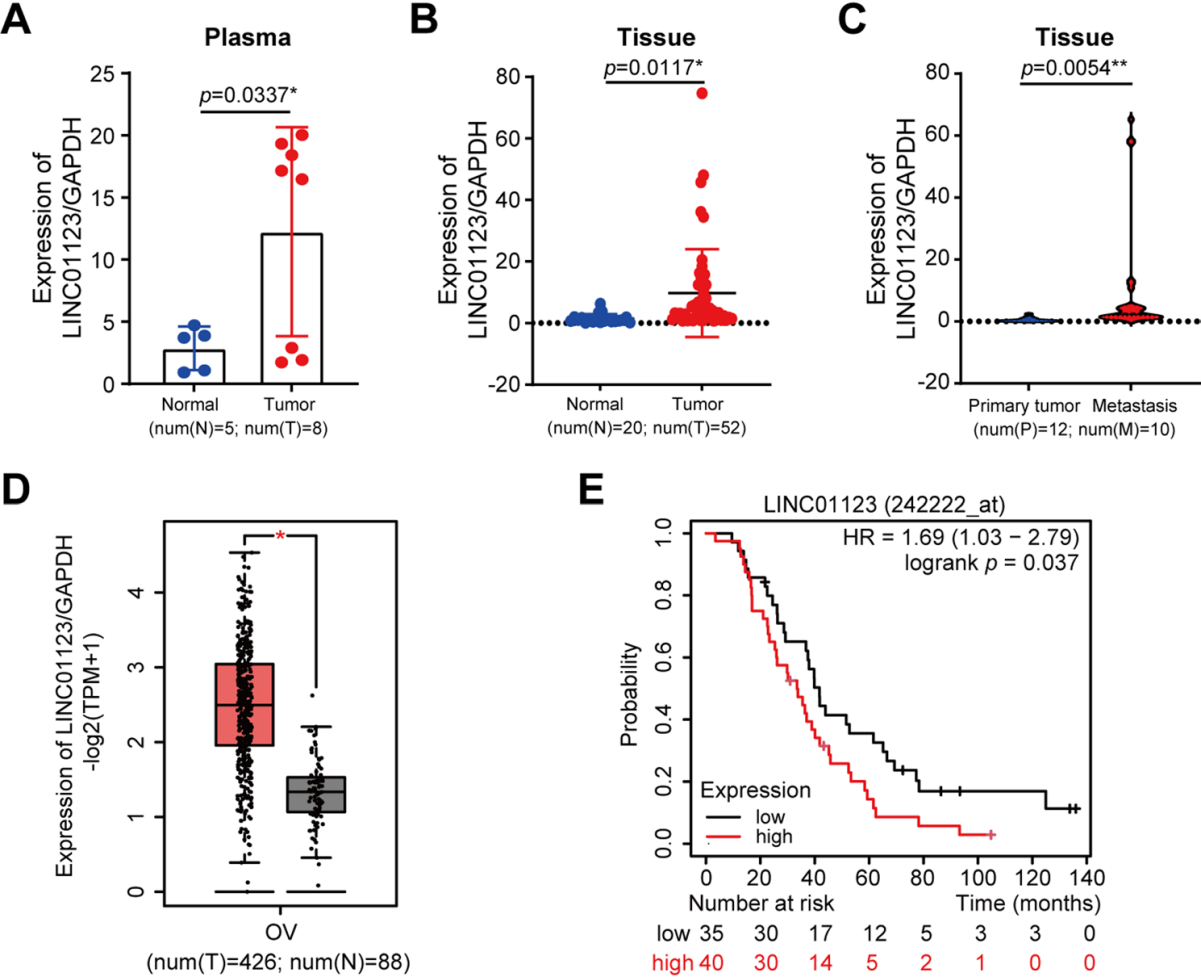
Differential expression of LINC01123 in OC patients (**A**) qPCR analysis of LINC01123 expression in OC plasma. **B** qPCR was used to detect LINC01123 expression in the non-tumoral tissues and OC tissue. **C** qPCR detection of LINC01123 expression in primary and metastatic OC tissues. **D** LINC01123 expression in OC and normal samples from the TCGA dataset. **E** Kaplan-Meier survival curves were produced for high (red line) and low (black line) LINC01123 expression in the OC samples. Statistical comparisons between two groups were performed using an unpaired Student’s t-test (**A-C**). Each dot represents an individual sample. Data are shown as the mean ± SD. *p < 0.05 or **p < 0.01

To validate our in vitro findings, we characterize the expression of LINC01123 from OC patients. First, we sought to determine whether there is circulating LINC01123 in the blood of patients with ovarian tumors. Thus, circulating RNA was extracted from diagnosed OC patients and healthy controls, and subsequent qPCR analysis for LINC01123 revealed that its expression level was significantly elevated in the plasma of patients compared to healthy controls (Fig. 3A). Next, we evaluated LINC01123 expression levels in tumor samples and in normal ovarian samples by qPCR. We detected high levels of LINC01123 expression in ovarian tumor samples, while it was almost undetectable in normal tissues (Fig. 3B). Likewise, the expression of LINC01123 was found to be higher in metastatic OC tissues than in primary OC tissues (Fig. 3C). We posit that the expression of LINC01123 may be related to carcinomatosis and consequently the worst prognosis. To test this hypothesis, we used the GEPIA2 data site (http://gepia2.cancer-pku.cn/#index) to determine the expression levels of LINC01123 in ovarian tumors and healthy controls. As shown in Fig. 3D, similar to those described in our small cohort, we observed significantly higher levels of LINC01123 in ovarian tumor samples compared to normal tissues (Fig. 3D). Furthermore, the expression levels of LINC01123 correlate with a bad prognosis (http://kmplot.com/analysis/) (Fig. 3E).

### LINC01123 encodes a micropeptide, YG-6

**Fig. 4:**
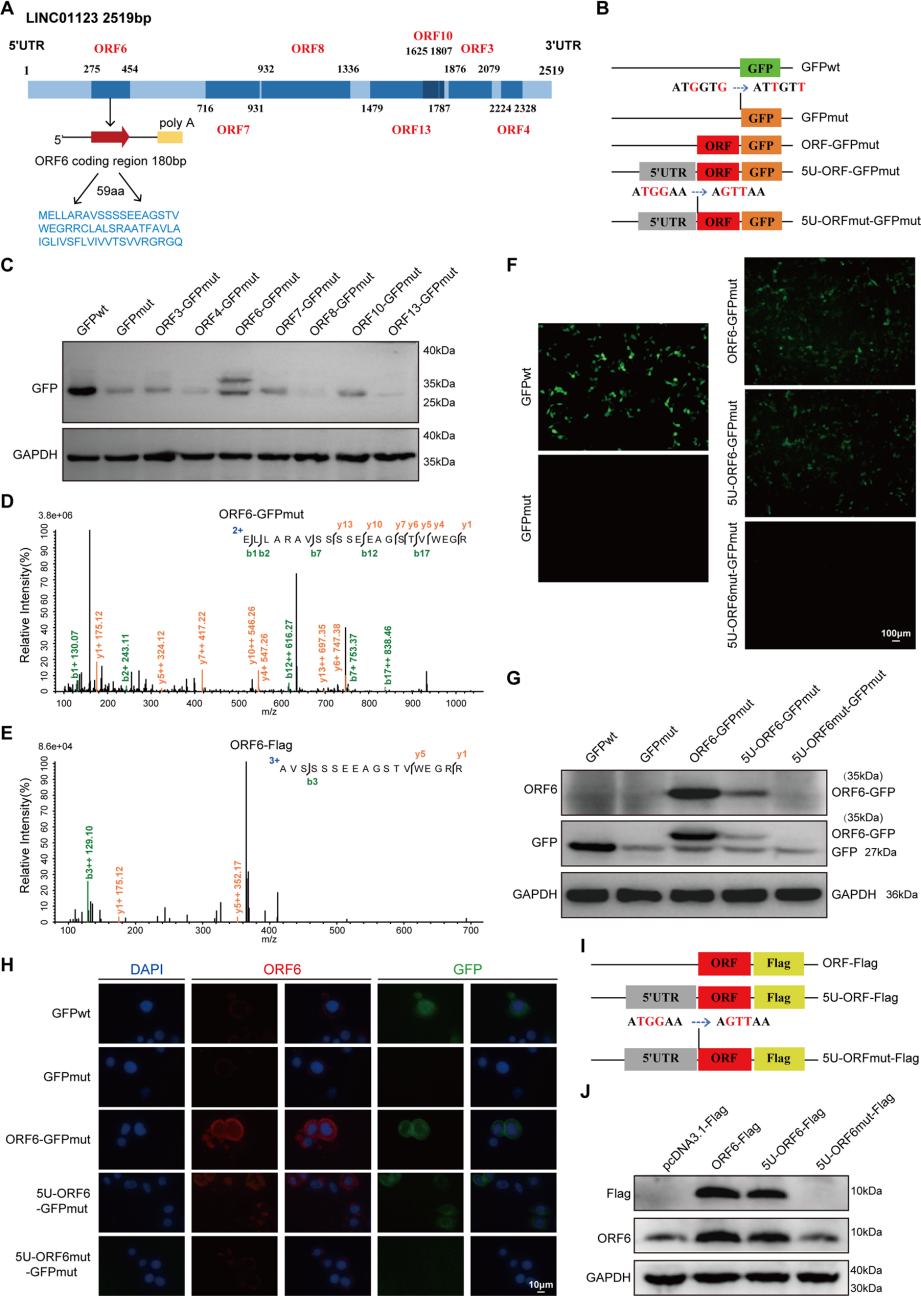
LINC01123 encodes a micropeptide, YG-6. **A** Locations of seven predicted ORFs of LINC01123 (ORFs longer than 30 aa’s in the sense (+) orientation were predicted and the ORF of the repeating sequence is removed). **B **Diagram of the GFP fusion constructs used for transfection. The start codon ATGGTG of the GFP (GFPwt) gene is mutated to ATTGTT (GFPmut). The start codon ATGGAA of the ORF6 is mutated to AGTTAA. **C** The 7 ORF-GFP fusion constructs were transfected into OC cells. Each ORF-GFP fusion protein was detected by WB using anti-GFP antibody. **D**-**E** The peptides in the ectopically expressed ORF6-GFP (**D**) and ORF6-Flag (**E**) were identified using MS. **F** The indicated constructs were transfected into HO-8910 cells for 48 h, and the GFP fluorescence was detected using a fluorescence microscope (Scale bars: 100 μm). **G** The indicated constructs were transfected into HO-8910 cells for 48 h, ORF6-GFP fusion protein levels were determined by WB with anti-GFP and ORF6 antibodies. **H** Immunofluorescence (IF) staining of the ORF6 peptide with an anti-ORF6 antibody and detection of GFP expression. Scale bars: 10 μm. **I** Schematic of the ORF6 and Flag tag fusion construct. **J** The levels of ORF6-Flag fusion peptide were detected by performing WB with anti-Flag and anti-ORF6 antibodies. The experiments were performed in triplicate (**C**, **F**-**H**, **J**)

Since the bioinformatic analysis predicted that LINC01123 may encode a peptide (Fig. 2C), we selected ORFs whose length is all greater than 30 aa in the sense (+) orientation with an initiating ATG codon, and after excluding ORFs with sequence coincidence, we identified 7 ORFs with potential encoding function (Fig. 4A). We fused each ORF to the N-terminus of the start codon-mutated GFP vector (GFPmut, where ATGGTG is mutated to ATTGTT) to determine whether the ORF could initiate the re-expression translation of the start codon-mutated GFP (Fig. 4B). WB analysis revealed that only LINC01123-ORF6-GFPmut was able to be translated (Fig. 4C). Consistently, fluorescence imaging corroborated that the green fluorescent signal was exclusively restored in the LINC01123-ORF6-GFPmut group (Fig. S4A). ORF6 is the first ORF located on the full length of LINC01123 (Fig. 4A), which is consistent with previous reports indicating that the first ORF at the 5’UTR of the sense strand is most likely to be translated, and that the start codon closer to the 5’UTR has a stronger ability to initiate translation due to its greater affinity for ribosome binding [33, 34]. These findings indicate that only ORF6 is capable of initiating the expression and translation of the mutant GFP and encoding it as a micropeptide. Then, the ORF6-GFPmut fusion construct was transfected into OC cells and the ORF6-GFP fusion peptide was immunoprecipitated using an anti-GFP antibody. The unique peptide fragments in the ORF6 peptide were identified using mass spectrometry (MS) (Fig. 4D). Similarly, the unique peptide fragments of ORF6 peptide were also identified by MS in the ORF6-Flag fusion construct (Fig. 4E).

To further validate that ORF6 has an encoding function, we mutated the start codon of ORF6 and observed GFP expression. We generated a series of GFPmut-fused plasmids containing the ORF6-GFPmut, the 5’-untranslated region (5U-ORF6-GFPmut), and 5U-ORF6mut-GFPmut (the start codon of ORF6 is mutated, where ATGGAA is mutated to AGTTAA) (Fig. 4B). Substantial expression of the ORF6-GFPmut fusion protein was observed in ORF6-GFPmut and 5U-ORF6-GFPmut-transfected cells (Fig. 4F), whereas the mutation of the start codon of the ORF6 (5U-ORF6mut-GFPmut) abolished the translation of the predicted ORF6 in LINC01123 (Fig. 4F). To further investigate the translation of ORF6, we generated an antibody specific to ORF6, the expression of the ORF6-GFPmut fusion protein was not detected by the anti-ORF6 antibody when the start codon ATG of ORF6 was mutated (Figs. 4G-H). These results demonstrate that the start codon of ORF6 is functional and capable of initiating the translation of GFPmut, whereas this activity is abolished upon its mutation. We next constructed ORF6-derived plasmids carrying a small Flag-tag (schematic diagram Fig. 4I). Subsequent WB analysis revealed that fusion bands of ORF6 were readily detectable with both anti-Flag and anti-ORF6 antibodies. However, the ORF6 fusion peptide became undetectable when its start codon was mutated (Fig. 4J). Similar results were also observed in flow cytometry analysis (Fig. S4B). Collectively, these data suggest that LINC01123, which was previously annotated as lncRNA, actually encodes a 59 aa micropeptide and promotes OC cell migration, so we named it YG-6 (LINC01123 Yield peptide Gaining metastasis function: open reading frame no.6).

### The YG-6 peptide is endogenously, naturally produced, and upregulated in OC

**Fig. 5:**
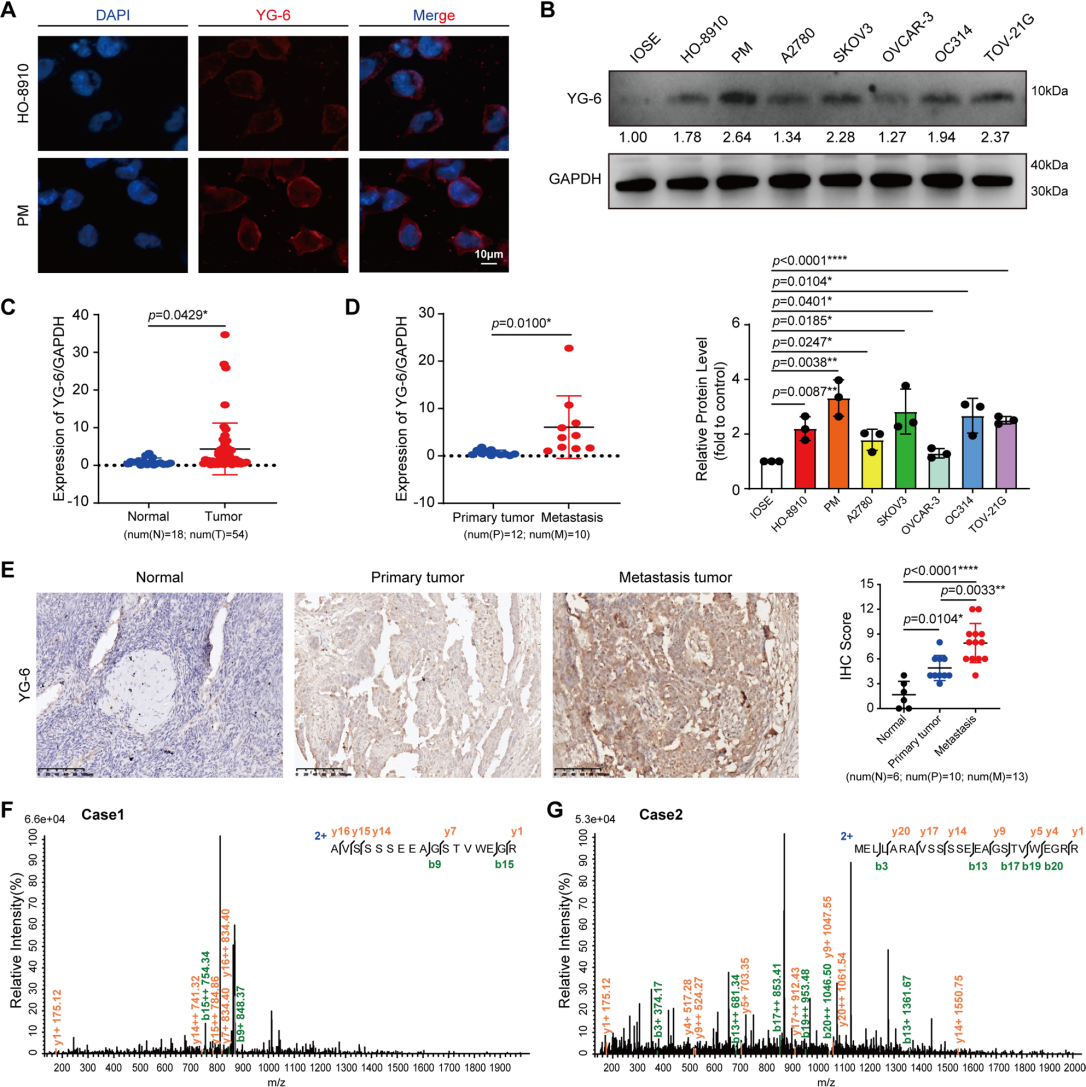
The YG-6 peptide is endogenously, naturally produced, and upregulated in OC. **A** The YG-6 peptide was immunostained with anti-YG-6 antibodies in PM and HO-8910 cells. Nuclei were stained with DAPI (blue). Scale bar: 10 μm. **B** WB was used to detect YG-6 peptide expression in human normal ovarian epithelial cells (IOSE) and OC cells. **C**-**D** Relative mRNA expression of YG-6 was determined by qPCR comparing non-tumor versus tumor tissues (**C**) and primary tumor versus metastatic lesions (**D**). Each dot represents an individual clinical sample. **E** Representative IHC images showing YG-6 peptide expression: non-tumor tissues, primary OC tissue, metastatic OC tissue. Scale bar: 100 μm. The final IHC score (H-score) was calculated by multiplying the intensity score by the percentage score (range: 0–12). **F**-**G** The unique peptide in the endogenous, natural YG-6 in tumor tissues was identified using MS. Statistical significance was determined by an unpaired Student’s t-test for comparisons between two groups (**C**-**D**), or one-way ANOVA followed by Dunnett’s (**B**) or Tukey’s post-hoc (**E**) test for comparisons among multiple groups. Each experiment was performed in triplicate. The data are represented as the means ± SD. *p < 0.05, **p < 0.01, ***p < 0.001, or ****p < 0.0001

Sequence alignment revealed no matching proteins and known domains/motifs in YG-6, indicating that it is an uncharacterized peptide. We detected the YG-6 peptide using anti-YG-6 antibodies to validate the presence and expression of the natural, endogenous YG-6 peptide in cells and tissues. IF (Fig. 5A), WB (Fig. 5B), immunohistochemistry (IHC) assay (Fig. 5E), and MS analysis (Figs. 5F-G) confirmed the presence of natural endogenous YG-6 peptide in OC cells and tissues. IF results showed a higher expression level of YG-6 in PM cells than in HO-8910 cells (Fig. 5A). Consistently, YG-6 levels were also substantially upregulated in the OC cells compared with the normal ovarian epithelial cell (Fig. 5B). Additionally, the expression of YG-6 in tissues was detected by qPCR. The results showed that YG-6 expression was significantly higher in OC tissues than in normal tissues (Fig. 5C), and it was further elevated in metastatic OC tissues compared to primary lesions (Fig. 5D). Furthermore, IHC confirmed elevated YG-6 expression in ovarian tumor tissues relative to non-tumor tissues, with even higher levels observed in metastatic lesions (Fig. 5E). More importantly, the unique peptide fragments in the natural, endogenous YG-6 peptide were identified using MS analysis **(**Figs. 5F-G**)**, further indicating that the natural, endogenous YG-6 peptide was present in OC tissues. Taken together, these data suggest that YG-6 is endogenously, naturally produced in human cells and tissues.

### YG-6, but not LINC01123 itself, promotes OC cell migration and tumor metastasis *in vitro* and *in vivo*

**Fig. 6:**
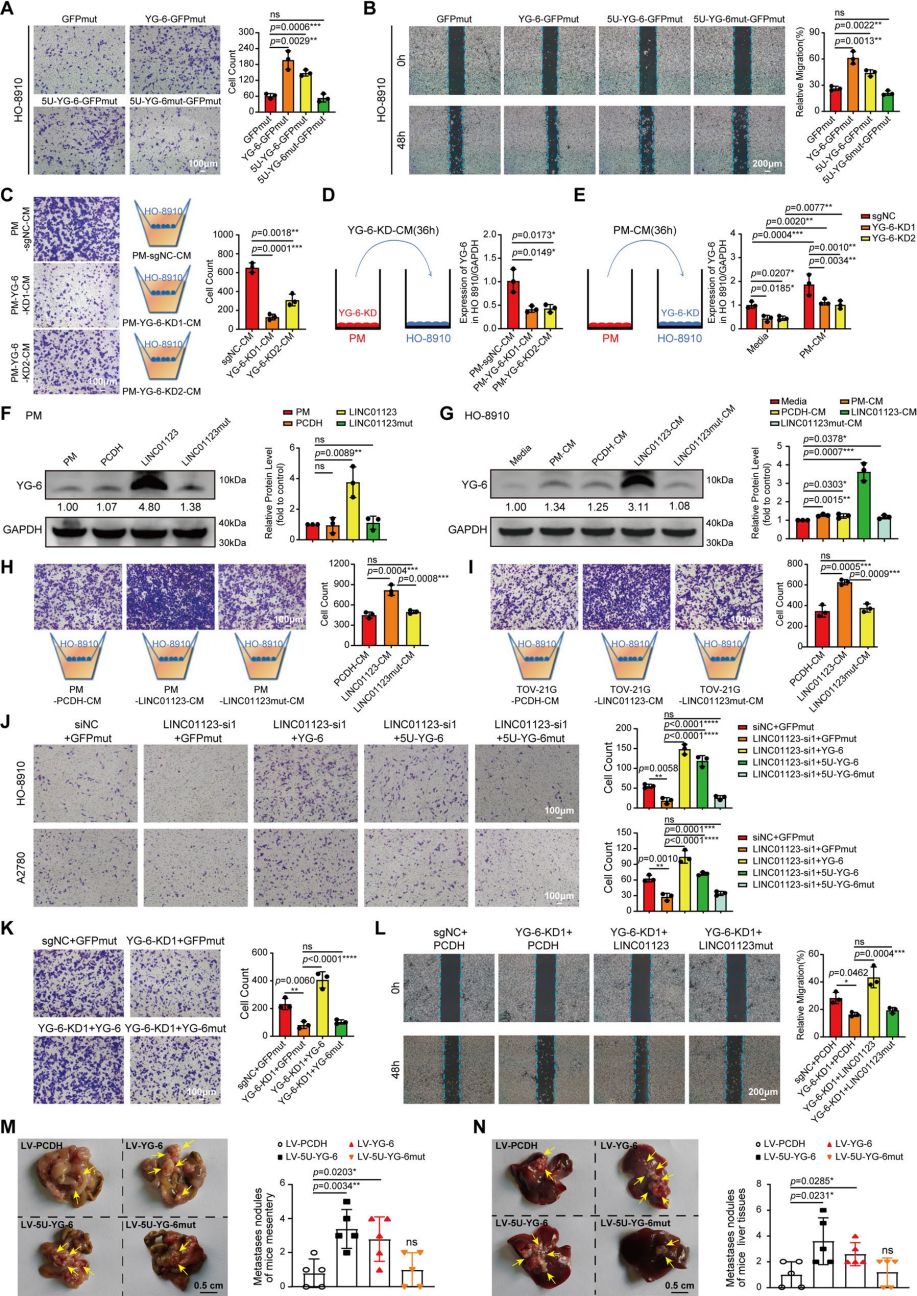
YG-6, but not LINC01123 itself, promotes OC cell migration and tumor metastasis in vitro and in vivo. **A**-**B** HO-8910 cells were transfected with YG-6, 5U-YG-6, and 5U-YG-6mut, the migration ability was determined by Transwell (**A**) and wound healing assays (**B**). Scale bar: 100 μm (**A**) and 200 μm (**B**). **C** Representative images of cell migration in HO-8910 were co-cultured with PM^YG−6−KD^. Scale bar: 100 μm (Left). The number of cells was counted by ImageJ (Right). **D** YG-6 knockdown in PM cells, collection of corresponding CM (36 h), co-incubation with HO-8910 (24 h), and detection of YG-6 uptake by HO-8910. **E** YG-6 was knocked down in HO-8910, and PM-CM (36 h) was collected, and then incubated with the corresponding 8910 well for 24 h to detect the uptake of YG-6 in HO-8910. **F**-**G** WB was used to determine the expression of YG-6 in different treatment groups. **H**-**I** Representative images of cell migration in HO-8910 were co-cultured with PM^LINC01123/LINC01123mut^ and TOV-21G^LINC01123/LINC01123mut^. Scale bar: 100 μm (Left). The number of cells was counted by ImageJ (Right). **J** The effect of co-transfection with YG-6, 5U-YG-6, and 5U-YG-6mut after silencing LINC01123 on different OC cell migration abilities was detected by Transwell. Scale bar: 100 μm (Left). The number of cells was counted by ImageJ (Right). **K** Co-transfecting YG-6-related plasmids with YG-6 KD1 into OC cells, and detecting their migration by the Transwell assay. Scale bar: 100 μm (Left). The number of cells was counted by ImageJ (Right). **L** Co-transfecting LINC01123-related plasmids with YG-6 KD1 into OC cells, and the migration was determined by the wound healing assay. Scale bar: 200 μm. **M**-**N** Representative images (n = 5 mice per group, Left) and metastatic nodule plots (Right) of mice mesentery (**M**) and liver tissues (**N**). Scale bar: 0.5 cm. Each experiment was performed in triplicate. Statistical significance was determined by one-way ANOVA followed by Dunnett’s or Tukey’s post-hoc test (**A**-**N**) for comparisons among multiple groups. The data are represented as the means ± SD. *p < 0.05, **p < 0.01, ***p < 0.001, or ****p < 0.0001, ns indicates no significance

To investigate the influences of the YG-6 peptide and LINC01123 on tumor progression, the YG-6, 5U-YG-6, and 5U-YG-6mut constructs, which all contained GFP tags, were transfected into OC cells. Transwell and wound healing assay showed that when both YG-6 and 5U-YG-6 constructs expressed YG-6 peptide, it can significantly promote the migration of OC cells (Figs. 6 A-B). In contrast, the 5U-YG-6mut, which contains a mutated YG-6 start codon and therefore does not encode the YG-6 peptide, did not alter OC cell migration (Figs. 6 A-B). This indicates that the start codon of YG-6 can also be recognized functionally. We also excluded the possible changes in migration ability caused by cell proliferation. CCK-8 (Fig. S5A), flow cytometry (Fig. S5B), and clone formation assay (Fig. S5C) all showed that YG-6 could not promote OC cell proliferation. Additionally, we observed that the suppression of YG-6 in PM cells with high expression of YG-6 notably inhibited the migration of OC cells (Figs. S6A-B). Subsequently, YG-6 was knocked down in PM cells, and the collected conditioned medium was used to co-culture HO-8910 cells. The results showed that, compared with the control, this medium not only significantly inhibited the migratory capacity of HO-8910 cells (Fig. 6C) but also markedly weakened their ability to take up YG-6 (Fig. 6D). Similarly, an analogous effect was observed in TOV-21G, an OC cell line with distinct molecular characteristics (Figs. S6C-D).

We also knocked down YG-6 in HO-8910 cells, afterwards, PM-CM (36 h) was collected at the same time and incubated with the corresponding HO-8910 well for 24 h. It was found that PM-CM could also up-regulate YG-6 expression in HO-8910 (Fig. 6E). In addition, after transfecting the plasmids LINC01123 and LINC01123mut (LINC01123 with mutated YG-6 start codon) in PM, the results showed that LINC01123mut did not affect the transcription level of LINC01123 (Fig. S6E). However, YG-6 protein expression was markedly reduced in the LINC01123mut construct (Fig. 6F). Finally, 4 groups of supernatant were collected and then co-incubated with HO-8910, Media (1640) was used as the control group. WB was used to detect YG-6 expression in HO-8910, and it was found that compared with the control group, YG-6 expression was significantly up-regulated in the LINC01123 overexpression group (Fig. 6G), whereas this effect was not observed in the LINC01123mut group (Fig. 6G). These results suggest that exosomal LINC01123 derived from HMOs can increase YG-6 expression in recipient cells. Subsequently, PM cells were transfected with LINC01123 and LINC01123mut (LINC01123 with mutated YG-6 start codon), and the conditioned medium was collected to co-culture with HO-8910 cells. The results showed that, compared to the control, LINC01123 overexpression significantly promoted the migration of HO-8910 cells, whereas this promigratory effect was markedly attenuated in the LINC01123mut group (Fig. 6H). Similarly, an analogous result was observed in TOV-21G cells (Fig. 6I). These results indicate that the pro-migratory function of LINC01123-Exos derived from HMOs is primarily mediated by the YG-6 peptide encoded by LINC01123, rather than by the LINC01123 RNA molecule itself.

Additionally, WB analysis showed that YG-6 expression was detectable upon overexpression of both YG-6 and LINC01123 in HO-8910 cells. In contrast, its expression was markedly reduced in the LINC01123mut group, where the start codon of YG-6 was mutated (Fig. S6F). Further functional experiments showed that both YG-6 and LINC01123 were able to promote cell migration, while these effects were attenuated in LINC01123mut-transfected cells (Fig. S6G). Next, we used LINC01123-siRNA to knock down the expression of LINC01123 in OC cells, and in these cells, we restored the expression of YG-6, 5U-YG-6, and 5U-YG-6mut. Transwell (Figs. 6J, S6I) and wound healing assay (Fig. S6H) found that knocking down LINC01123 inhibited the migration of OC cells, these changes returned to control levels after overexpression of YG-6, but not in the 5U-YG-6mut group. These results suggest that YG-6 peptide rather than LINC01123 RNA itself promoted progression. YG-6-KD1, which has a better knocking effect, is chosen for further functional experiments. Therefore, we transfected YG-6, 5U-YG-6, and 5U-YG-6mut in YG-6-KD1 cell strains, and found that while re-expression of wild-type YG-6 significantly restored the migratory capacity of these cells, the non-coding mutant (5U-YG-6mut) failed to elicit any such rescue effect (Fig. 6K). Similarly, transfection of YG-6-KD1 suppressed cell migration, and the capability of cell migration could be restored by overexpression of LINC01123 transcript, while LINC01123mut failed to rescue this phenotype (Fig. 6L). These results suggest that LINC01123 promoted OC metastasis mainly through oncopeptide YG-6.

We finally examined the role of YG-6 in OC metastasis in vivo, the stable overexpression OC cell lines with the indicated YG-6 were constructed, and after the overexpression effect (Fig. S7A) and migration function (Fig. S7B) were verified, intraperitoneally (i.p.) injected nude mice, then killed 30 days later, and the number of metastatic nodules was observed. In vivo experiments also confirmed that YG-6 can promote OC metastasis, but 5U-YG-6mut lost this effect (Figs. 6M-N). The global landscape of tumor formation via intraperitoneal (i.p.) injection in nude mice is shown in Fig. S7C.

### YG-6 peptide interacts with ACTC1 protein

**Fig. 7:**
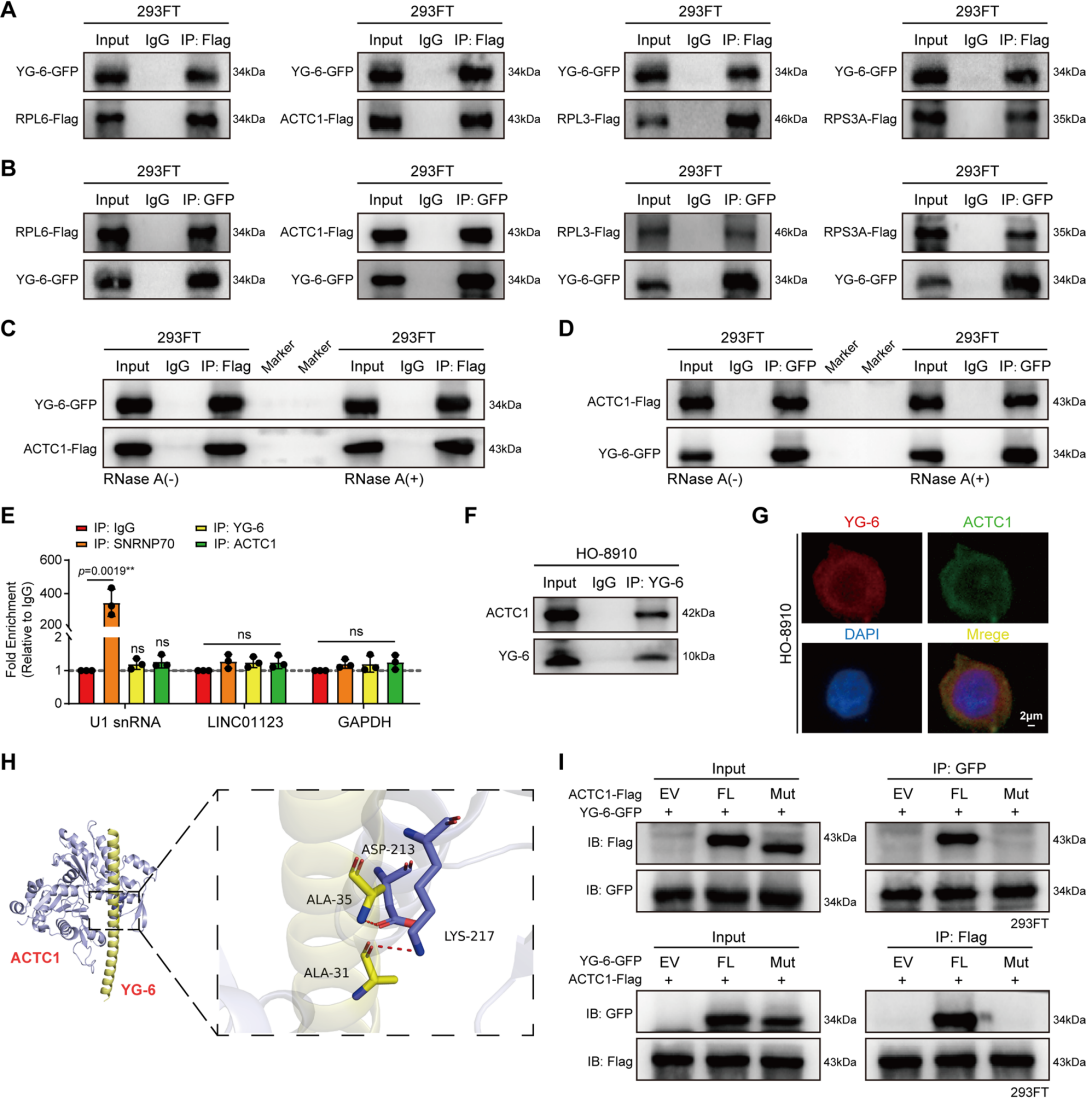
YG-6 peptide interacts with ACTC1 protein. **A**-**B** Exogenous Co-IP detected the binding of YG-6 to top genes in the focal adhesion signaling pathway. **C**-**D** The ACTC1-Flag and YG-6-GFP vectors were co-transfected into HEK 293FT cells, ACTC1-Flag and YG-6-GFP complexes were Co-IPed with anti-Flag and anti-GFP antibodies in the absence (**C**) or presence (**D**) of RNase A (20 µg/mL) treatment, and YG-6-GFP and ACTC1-Flag YG-6-GFP were detected, respectively. **E** RIP assay was performed in HO-8910 cells. The results showed that LINC01123 did not significantly enrich with either YG-6 or ACTC1 protein. SNRNP70 antibody and U1 snRNA were used as positive controls to ensure the validity of the RIP system. **F** Endogenous Co-IP analysis of the interaction between YG-6 and ACTC1 in HO-8910 cells. Whole-cell lysates were immunoprecipitated with an anti-YG-6 specific polyclonal antibody, followed by immunoblotting with anti-ACTC1 and anti-YG-6 antibodies. IgG served as a negative control. **G** IF staining showing the subcellular localization of YG-6 (red) and ACTC1 (green) in HO-8910 cells. Nuclei were stained with DAPI (blue). Scale bar: 2 μm. **H** Molecular docking model predicting the binding interface between the YG-6 micropeptide (yellow) and ACTC1 protein (light blue). The critical residues involved in the interaction include ALA-31, ALA-35, ASP-213, and LYS-217. **I** Exogenous Co-IP assay in 293FT cells co-transfected with Flag-tagged ACTC1 (FL or Mut) and GFP-tagged YG-6 (FL or Mut). Data are presented as the mean ± SD from at least three independent experiments (**E**). Statistical significance was determined using one-way ANOVA followed by Tukey’s post-hoc test. Each experiment was performed in triplicate. *p < 0.05, **p < 0.01, ***p < 0.001, or ****p < 0.0001, ns indicates no significance

The YG-6 peptide is an uncharacterized micropeptide with a lack of homology to other proteins. Based on the significant tumor-promoting role of YG-6 in OC, we further explored its regulation mechanism. First, we performed coimmunoprecipitation on cell lysates with anti-Flag antibody and quantified the proteins specifically enriched in the immunoprecipitation complex via MS, and performed silver staining (Fig. S8A). The results showed that 99 proteins that interact with YG-6 peptide were identified (Table S4). To characterize the functional roles of these YG-6 interacting proteins, we use bioinformatics to analyze the signaling pathway processes in which they are involved. Gene Ontology (GO) annotation assay showed that most of the proteins that interact with the YG-6 peptide significantly accumulate focal adhesion, a signaling pathway closely related to migration, suggesting that YG-6 may participate in the regulation of OC development by interacting with adhesion factors (Fig. S8B). The first 4 genes (RPL6, ACTC1, RPL3, RPS3A) were preferred in Focal adhesion, and we further confirmed that YG-6 can interact with these proteins by Co-IP experiments (Figs. 7A-B). Functional experiments show that ACTC1 can significantly restore the phenotype of YG-6 knockdown inhibiting OC migration (Fig. S8C). Therefore, we here select ACTC1 for further investigation.

We further confirmed that the interaction between YG-6 and ACTC1 persisted both in the presence and absence of RNase A treatment, indicating that their binding is RNA-independent (Figs. 7C-D). This was further supported by an RNA immunoprecipitation (RIP) assay, which revealed that LINC01123 did not bind to either the YG-6 micropeptide or the ACTC1 protein (Fig. 7E). To confirm this direct protein-protein interaction at the endogenous level, we performed a Co-IP assay in HO-8910 cells using a specific polyclonal antibody against YG-6. The results showed that the YG-6 antibody successfully pulled down endogenous ACTC1 protein (Fig. 7F). Furthermore, immunofluorescence assays demonstrated that YG-6 and ACTC1 were predominantly colocalized in the cytoplasm and on the cell membrane (Fig. 7G). Taken together, these data indicate that the YG-6 peptide directly interacts and colocalizes with ACTC1 in a manner that is independent of RNA. Subsequently, molecular docking simulation predicted the binding sites between the two proteins, identifying ALA-31, ALA-35, ASP-213, and LYS-217 as key binding residues (Fig. 7H). To verify this prediction, we constructed mutants with deletion mutations at these specific sites. Exogenous Co-IP results showed that, compared with the full-length (FL), the binding capacity between YG-6 and ACTC1 was significantly reduced or abolished following mutation (Mut) of the specific binding regions (Fig. 7I). These data collectively confirm that the YG-6 micropeptide binds directly to ACTC1.

In addition, the expression of ACTC1 in OC tissues was higher than that in non-tumor tissues by WB detection (Fig. S9A). At the same time, we also analyzed ACTC1 with some GEO datasets (GSE7305, GSE26712, and GSE26193), and found that ACTC1 was highly expressed in OC tissues and was associated with poor prognosis of OC (Figs. S9B-D). The area under the ROC curve of different GEO datasets (GSE26193 and GSE63885) also indicated that ACTC1 was a significant independent prognostic factor (Figs. S9E-G). Furthermore, the GSE26193 dataset revealed higher ACTC1 expression in patients with high-grade OC (Fig. S9H). Collectively, these results indicate that ACTC1 is overexpressed in OC and is closely associated with an unfavorable prognosis, suggesting its potential as a molecular target.

### YG-6 binds ACTC1 to participate in the focal adhesion signaling pathway to regulate the biological behavior of OC

**Fig. 8:**
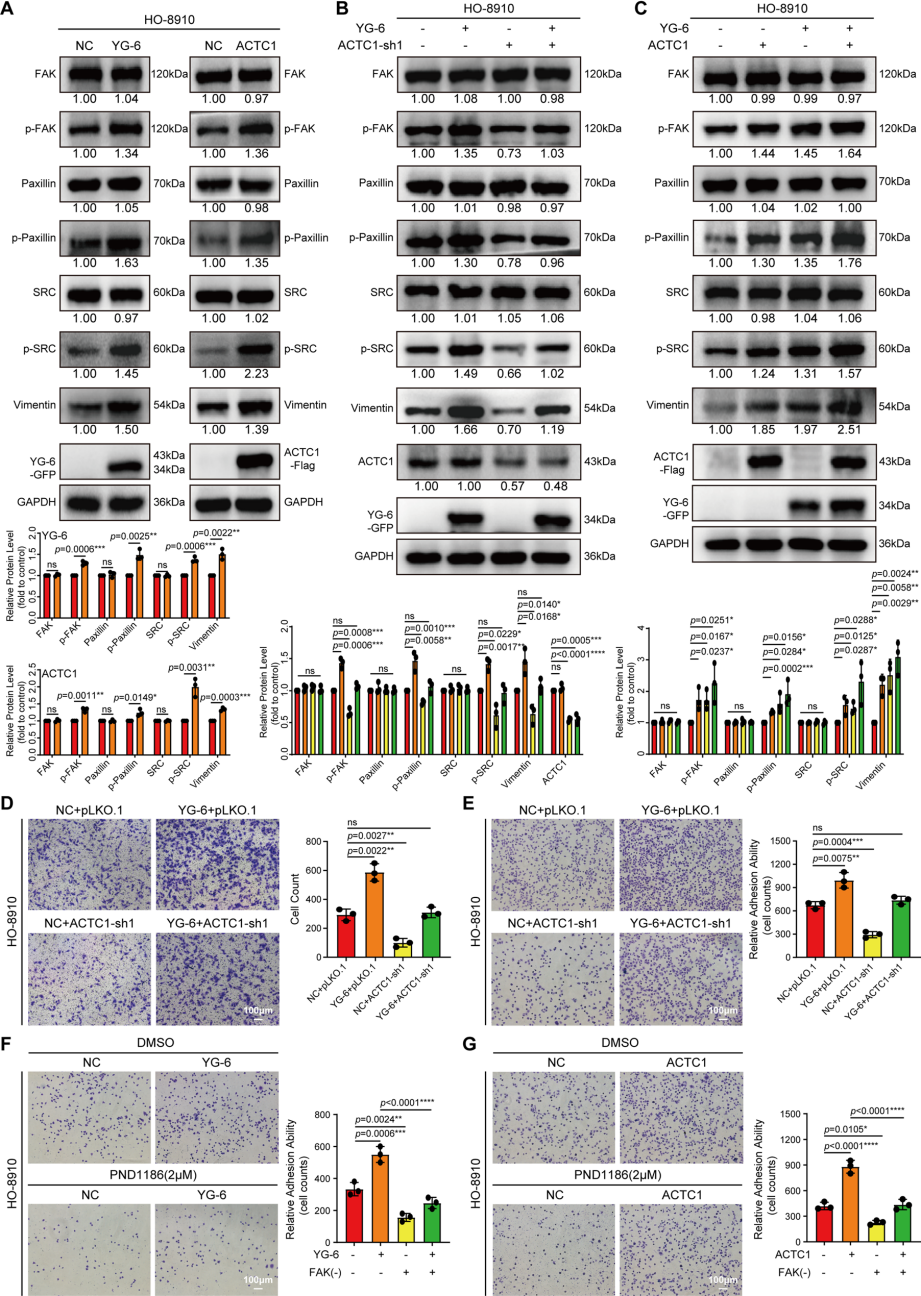
YG-6 binds ACTC1 to participate in the focal adhesion signaling pathway to regulate the biological behavior of OC. **A** WB analysis of the focal adhesion signaling pathway markers in HO-8910 cells following over-expression of YG-6 (Left) or ACTC1 (Right). **B** WB analysis of signaling pathways in YG-6-overexpressing cells with ACTC1 knockdown. HO-8910 cells were co-transfected with YG-6 expression vectors and either ACTC1-sh or a negative control shRNA. Protein levels of focal adhesion signaling pathway markers were determined at 48 h post-transfection. **C** WB analysis of signaling pathways following co-overexpression of YG-6 and ACTC1. HO-8910 cells were transfected with YG-6 and ACTC1 expression plasmids individually or in combination. Protein levels of focal adhesion signaling pathway markers were determined at 48 h post-transfection. **D**-**E **Co-transfected with YG-6 and either ACTC1-sh1 or a negative control (pLKO.1) in HO-8910 cells to assess migratory (**D**) and adhesion capacity (**E**). Scale bar: 100 μm. **F**-**G** HO-8910 cells were transfected with YG-6 (**F**) or ACTC1 (**G**) and subsequently treated with the FAK inhibitor PND1186 (2 µM) or DMSO control. Cell adhesion capacity was evaluated by counting adherent cells per field. Scale bar: 100 μm. Quantitative analysis of protein levels (normalized to GAPDH) based on gray value densitometry from three independent experiments (**A**-**C**). The data are represented as the means ± SD. Statistical significance was determined using one-way ANOVA followed by Dunnett’s or Tukey’s post-hoc test (**A**-**G**). Each experiment was performed in triplicate. *p < 0.05, **p < 0.01, ***p < 0.001, or ****p < 0.0001, ns indicates no significance

The interacting proteins enriched with YG-6 above are mainly involved in focal adhesion, so we detected the expression of relevant indicators by WB, and we could see that the phosphorylation levels of FAK, Paxillin, and SRC were up-regulated, supporting the regulation of focal adhesion signaling cascade by YG-6 and ACTC1 **(**Figs. 8A, S10A). WB recovery experiments can also show that overexpression of YG-6 can activate the focal adhesion signaling pathway, and knocking down ACTC1 can inhibit the focal adhesion signaling pathway, but after co-transfection, knocking down ACTC1 can reverse the activation signal of YG-6 to a certain extent (Figs. 8B, S10B). In addition, when overexpression of YG-6 or ACTC1 alone promoted the focal adhesion pathway, the activation was more pronounced after co-transfection of both genes (Figs. 8C, S10C). Then, when YG-6 and ACTC1 are overexpressed, they can significantly change the morphology of OC epithelial cells from pebble-like to spindle-like, with loss of cell polarity and altered skeleton (Figs. S11A-B), thus enhancing migration ability and promoting cell adhesion (Figs. S11C-D). Transwell (Figs. 8D, S11E) and adhesion (Figs. 8E, S11F) assays further demonstrated that ACTC1 knockdown partially abrogated the pro-migratory and adhesion effects induced by YG-6 overexpression. Similarly, ACTC1 overexpression can also revert to YG-6 knockdown phenotypes that inhibit cell adhesion (Figs. S11G). PND1186 is an inhibitor of FAK, and we found that it can change the morphology of OC cells (Fig. S11H). Finally, we used the PND1186 and found that the adhesion ability of OC cells induced by YG-6 and ACTC1 overexpression was significantly inhibited (Figs. 8F-G, S11I-J). These results indicate that YG-6 stimulates OC progression mainly via ACTC1.

**Fig. 9:**
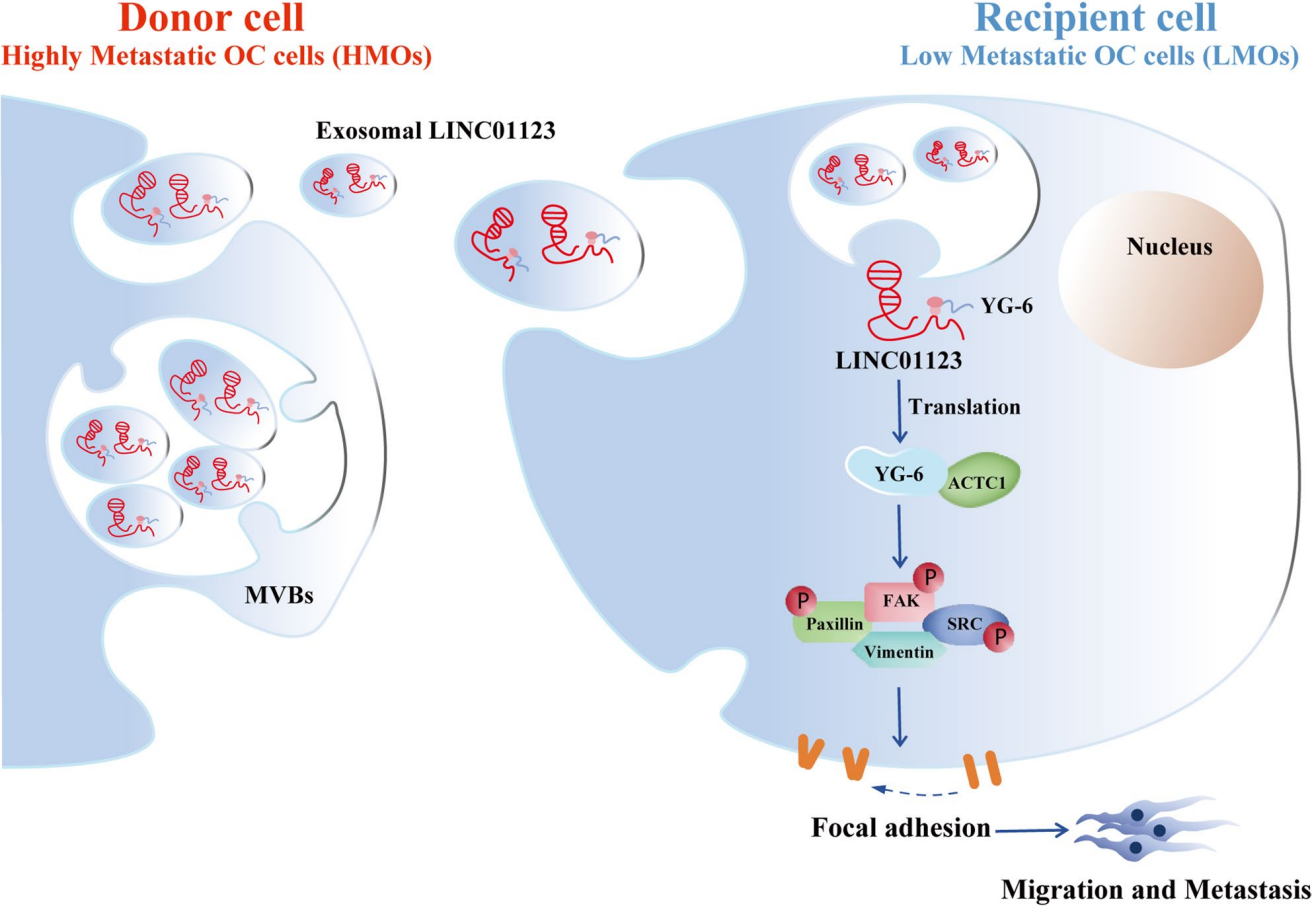
A working model describes the mechanism by which HMOs (Donor cells) reshaped the phenotype in LMOs (Recipient cells)

Our findings are depicted as a cartoon presented in Fig. 9. It is shown that the HMOs (highly migratory OC cells, Donor cells) released exosomes containing LINC01123, which reshaped the phenotype of the LMOs (low migratory OC cells, Recipient cells). Our multiple lines of evidence suggest that LINC01123 encodes the 59 aa micropeptide YG-6, which imparts carcinogenic effects to LINC01123 in OC. In addition, YG-6 interacts with ACTC1 to activate the focal adhesion signaling pathway that regulates cell migration and adhesion, thereby promoting the biological malignant behavior of OC.

## Discussion

Metastasis, causing about 90% deaths of cancer patients, is well known as the most significant characteristic of malignant cancer [2, 3]. Understanding the phenotypical intratumor heterogeneity is crucial for gaining insights into its diverse metastatic potential. OC, a gynecological malignancy with mortality second only to cervical cancer, and the clinical outcome is less than satisfactory [6, 35]. Due to the lack of specific symptoms and reliable biomarkers, most patients are diagnosed at an advanced stage [7]. Although treatment modalities have advanced with the introduction of targeted therapy and immunotherapy, the 5-year overall survival rate remains unsatisfactory, largely due to the difficulty in early diagnosis, high invasiveness, high rates of recurrence, and drug resistance [36–38]. Therefore, a comprehensive understanding of the molecular mechanisms underlying OC metastasis and the identification of novel prognostic biomarkers are of paramount importance.

Exosomes have been reported to mediate intercellular communication and act as potent promoters of cancer metastasis [39]. Exosomes are derived from multivesicular bodies, which are formed by almost all cell types. Tumor cells, however, typically secrete more exosomes than normal cells, with the quantity often correlating positively with tumor malignancy [40]. In our study, we employed OC cell lines with varying metastatic potentials to investigate the functional and mechanistic roles of exosomes in OC metastasis. First, extracellular vesicles were isolated from the conditioned medium of OC cells using differential centrifugation. Exosomes, with diameters ranging from 30 to 150 nm, were then characterized by TEM, NTA, and the presence of exosome markers Alix, HSP70, and TSG101. Subsequently, we observed that exosomes isolated from the medium of HMOs significantly enhanced the metastatic potential of LMOs. These results suggest that exosomes facilitate cell-cell interactions between cancer cells with different metastasis capabilities, which can accelerate the metastasis progression.

Accumulating evidence has shown that exosomal lncRNAs isolated from the conditioned media of donor cells can induce a series of cellular responses upon uptake by recipient cells [41, 42]. To identify the key functional factors involved in HMOs-PM exosomes, we performed RNA sequencing (RNA-Seq) on exosomes derived from high- and low-migratory OC cells, with a focus on lncRNA analysis. A total of 83 differentially expressed lncRNAs were screened. Through online prediction of lncRNA’s potential coding ability and functional screening experiments, we ultimately identified LINC01123. We performed the qPCR assay to assess LINC01123 expression in exosomes derived from the HMOs and LMOs. The results aligned with the Exos-RNA-Seq data, showing that LINC01123 was highly expressed in HMOs. Further analysis revealed that its encoded micropeptide, YG-6, significantly enhanced the migration ability of LMOs. Additionally, LINC01123 expression also significantly influenced the migratory capacity of OC cells. It is noteworthy that, in HMOs, mutation of the YG-6 start codon within the full-length LINC01123 transcript markedly attenuated its effect on promoting LMOs migration in the co-culture system. These results establish that the pro-migratory effect of LINC01123-Exos derived from HMOs is directly mediated by the YG-6 micropeptide, independent of the LINC01123 RNA molecule itself.

Recent advances in bioinformatics and biochemical methodologies have demonstrated that lncRNAs may encode functional peptides through short open reading frames (sORFs) [43–45]. With the evolution of ribosome profiling, MS analysis, and in silico prediction, several databases have been established to catalog micropeptides translated from noncanonical ORFs [46–48]. However, the specific functions and mechanisms of micropeptides remain poorly understood. In previous studies, due to the limitations of biological cognitive boundaries and the bottleneck of conventional techniques, the degree of research on lncRNAs encoding micropeptides and their contribution to living organisms has been ignored. Initial studies on lncRNA-encoded peptides primarily focused on plants and invertebrates [49, 50]. To date, only a few lncRNAs encoding peptides have been functionally characterized in the context of tumorigenesis and progression [51–55]. It is noteworthy that the absence of studies regarding OC regulation by lncRNA-encoded micropeptides remains evident.

Here, we have demonstrated and characterized the functions of a conserved endogenous protein YG-6 encoded by LINC01123 during OC progression in vitro and in vivo. In this study, we found that YG-6 can promote the migration ability of OC cells, and LINC01123 with mutated YG-6 start codon failed to regulate cell migration, indicating that YG-6 conferred the oncogenic effect to LINC01123 in OC. Therefore, YG-6 is further termed an oncopeptide. Multiple lines of experimental evidence indicate that YG-6 is present and highly expressed in both OC cells and tissues, demonstrating that it is endogenously, naturally produced in human cells and tissues. The inhibition of YG-6 can significantly suppress OC cell migration. Interestingly, we identified a total of 99 proteins interacting with YG-6 by MS. GO analysis showed that these proteins interacting with YG-6 significantly accumulated Focal adhesion, a signaling pathway closely related to migration, and Co-IP experiment confirmed that YG-6 could interact with ACTC1, and ACTC1 has also been reported to be closely related to tumor migration [56–58]. Both YG-6 and ACTC1 activated the Focal adhesion signaling pathway, and YG-6’s regulation of ovarian cancer cell migration and adhesion was largely dependent on ACTC1. Collectively, our findings suggest that YG-6 can bind to ACTC1, participate in the focal adhesion signaling pathway, and thereby regulate the biological behavior of OC.

Taken together, our current study demonstrates that the micropeptide YG-6, encoded by LINC01123 from HMOs-derived exosomes, is heavily internalized by LMOs and subsequently promotes their malignant behaviors. This finding suggests that exosome crosstalk between metastatic cancer cells represents a novel mechanism of cancer metastasis. Furthermore, our results reveal that LINC01123 exerts its function of promoting OC migration and adhesion by encoding a previously uncharacterized oncopeptide, YG-6, rather than functioning as an RNA molecule. This discovery underscores the therapeutic potential of targeting YG-6 in cancer treatment. Compared to large proteins, micropeptides are smaller and simpler in structure, offering unique advantages as therapeutic targets. Therefore, developing specific YG-6 inhibitors or blocking antibodies to validate this hypothesis in future studies would be worthwhile. Furthermore, the clinical applicability of YG-6 requires further validation of its prognostic value through large-scale clinical cohorts, as well as prospective studies to systematically evaluate its therapeutic efficacy.

Our research not only provides the first insights into the role of the novel micropeptide YG-6 in OC but also reaffirms the emerging view that lncRNAs can encode functional peptides, offering new perspectives on the function and mechanisms of lncRNAs.

## Conclusion

In summary, we identified a highly expressed lncRNA-LINC01123 in exosomes derived from HMOs, which encodes the micropeptide YG-6 and is internalized into LMOs in large quantities. Moreover, the oncopeptide YG-6 encoded by LINC01123 binds with ACTC1 to further regulate cell migration and adhesion, thus promoting the biological malignant behavior of OC cells (the working model is summarized in Fig. 9). These results suggest that the micropeptide YG-6 has great potential as a therapeutic target and tumor biomarker for OC.

## Supplementary Information

Supplementary Material 1.

Supplementary Material 2.

Supplementary Material 3.

Supplementary Material 4.

## Data Availability

No datasets were generated or analysed during the current study.

## Abbreviations

OC: Ovarian cancer
LncRNAs: Long non-coding RNAs
HMOs: Highly migratory OC cells
LMOs: Low migratory OC cells
YG-6: LINC01123 Yield peptide Gaining metastasis function-open reading frame no.6
Exos: Exosomes
CM: Conditioned medium
ORF: Open reading frame
qRT-PCR: Quantitative real-time polymerase chain reaction
siRNA: Small interfering RNA
GAPDH: Glyceraldehyde-3-phosphate dehydrogenase
TEM: Transmission electron microscopy
NTA: Nanoparticle tracking analysis
GO: Gene Ontology
GEO: Gene Expression Omnibus
DMEM: Dulbecco’s modified Eagle’s medium
WB: Western blotting
SDS-PAGE: Sodium dodecyl sulfate-polyacrylamide gel electrophoresis
FBS: Fetal bovine serum
PBS: Phosphate buffered saline
IHC: Immunohistochemistry
IF: Immunofluorescence
DAPI: 4’,6-Diamidino-2-phenylindole
Co-IP: Co-immunoprecipitation
BSA: Bovine serum albumin
CCK-8: Cell Counting Kit-8
FN: Fibronectin
MVBs: Multivesicular bodies
MS: Mass spectrometry
KLH: Keyhole Limpet Hemocyanin
CFA: Complete Freund’s Adjuvant
IFA: Incomplete Freund’s Adjuvant
CPC: Coding potential calculator
OS: Overall survival
UTR: Untranslated regions
RIP: RNA immunoprecipitation
FL: Full-length
Mut: Mutation
SD: Standard deviation
STR: Short tandem repeat
