# Supplementary material for: Micropeptide YG-6 encoded by exosomal LINC01123 derived from highly migratory ovarian cancer cells promotes tumor progression
Source: Mol Cancer. 2026 Mar 5;25:100. doi: 10.1186/s12943-026-02621-w (PMC13072579; doi:10.1186/s12943-026-02621-w)
Supplement: Supplementary file 1 — Supplementary Material 1. [file 12943_2026_2621_MOESM1_ESM.doc]

**Micropeptide YG-6 Encoded by Exosomal LINC01123 Derived from Highly Migratory Ovarian Cancer Cells Promotes Tumor Progression**

**Han Lei^1^, Zhengwei Zhou^1^, Chengyuan Li^1^,** **Lili Fan^4^, Qihan Wu^5^,** **Maonan Wang^1^, [Ke Guo](https://pubmed.ncbi.nlm.nih.gov/?term=Guo+K&cauthor_id=38265973)^3^,** **[Qiong Pan](https://pubmed.ncbi.nlm.nih.gov/?term=Pan+Q&cauthor_id=38265973)^2^, Gil Mor^6^, Guang Shu****^1*^, Juanni Li^1,8*^, Gang Yin^1,7,8*^**

^1^ Department of Pathology, Xiangya Hospital, Xiangya School of Basic Medical Sciences, Central South University, Changsha, Hunan, China.

^2^ Department of Obstetrics and Gynecology, The Third Xiangya Hospital of Central South University, Changsha, Hunan, China.

^3^ Department of Neurology, The Third Xiangya Hospital of Central South University, Changsha, Hunan, China.

^4^ Guangzhou Key Laboratory of Formula-Pattern of Traditional Chinese Medicine, School of Traditional Chinese Medicine, Jinan University, Guangzhou, Guangdong, China.

^5^ Shanghai-MOST Key Laboratory of Health and Disease Genomics, NHC Key Lab of Reproduction Regulation, Shanghai Institute for Biomedical and Pharmaceutical Technologies, Shanghai, China.

^6^ C.S. Mott Center for Human Growth and Development, Wayne State University, 275 E Hancock Av Detroit, MI, 48201 USA.

^7^ China-Africa Research Center of Infectious Diseases, Xiangya School of Basic Medical Sciences, Central South University, Changsha, Hunan, China.

^8^ National Clinical Research Center for Geriatric Disease (Xiangya Hospital), Central South University, Changsha, Hunan, China.

^*^ Corresponding author. Email: [gangyin@csu.edu.cn](mailto:gangyin@csu.edu.cn) (Gang Yin); [lijuanni2014@csu.edu.cn](mailto:lijuanni2014@csu.edu.cn) (Juanni Li); [shuguang78@csu.edu.cn](mailto:shuguang78@csu.edu.cn) (Guang Shu).**Supplementary Materials and Methods**

**Cell culture and transfection**

All cell lines used in this study included the human normal immortalized ovarian surface epithelial cell line IOSE and ovarian cancer (OC) cell lines (TOV-21G, OVCAR-3, OC314, A2780, and SKOV3) were purchased from the American Type Culture Collection (ATCC). The HO-8910 and PM cell lines were established in our laboratory through monoclonal screening and were strictly validated by Short Tandem Repeat (STR) profiling. These two cell lines were entirely consistent with the corresponding reference profiles in the consensus database. These cell lines were cultured in RPMI-1640 (BI) replenished with 10% fetal bovine serum (FBS) (BI), 100 µg/mL penicillin (Sigma) and 100 µg/mL streptomycin (Sigma). The human embryonic kidney (HEK)-HEK-293T/293FT was cultured in Dulbecco's Modified Eagle's Medium (DMEM) (BI) with 10% FBS. All cells were cultured at 37 ℃ in a humidified 5% CO_2_ incubator. Cells or conditioned media (CM) were collected for the experiment at the indicated time. 5× 10^5^ OC cells were seeded in a six-well plate and then reached 60-70% fusion degree per well, jetPRIME was applied for transient transfection of DNA and/or siRNA *in vitro* according to the manufacturer's instructions. These cells were digested and collected after 48 h of transfection for the subsequent assays. All of the sequences of primers are listed in **Table S1.**

**Exosome isolation and component extraction**

Exosomes were isolated and purified by ultracentrifugation from the OC cells-derived CM. Briefly, after 48 h of culture, the culture supernatant/fluid containing 2% exosome-depleted FBS was collected and centrifuged at 1,250 × g for 25 min at 4 °C, and then at 7,000 × g for 15 min at 4 °C to remove dead cells and cell debris. The supernatant was filtered by a 0.22 µm filter (Millipore, USA) and transferred to the ultracentrifuge tubes (Beckman Coulter, USA), then ultracentrifuged at 20,000 × g for 30 min at 4 °C to remove remaining macro polymers, the supernatant further ultracentrifuged at 110,000 × g for 1 h at 4 °C to collect vesicles (the size was smaller than 150 nm) and contaminated protein. Finally, the pellets were resuspended in ice-cold 1× PBS (110,000 × g for 1 h) to eliminate contaminated proteins and then resuspended in 1× PBS for further analysis. All steps were performed at 4 °C.

For RNA extraction, exosomal total RNA was isolated from the purified pellets using the TRIzol LS Reagent (Invitrogen, USA) according to the manufacturer's instructions. Briefly, TRIzol LS was added to the exosome suspension at a 3:1 ratio and homogenized. Following a 5 min incubation, chloroform was added (0.2 mL per 0.75 mL TRIzol LS) for phase separation. The mixture was centrifuged at 12,000 × g for 15 min at 4 °C. The upper aqueous phase was collected, and RNA was precipitated with an equal volume of isopropanol. After further centrifugation, the RNA pellet was washed with 75% ethanol, air-dried, and resuspended in RNase-free water. The concentration and purity of the isolated RNA were determined using a NanoDrop Spectrophotometer.

For protein analysis, the purified exosomal pellets were resuspended in RIPA lysis buffer (Beyotime, China) supplemented with a 1% protease and phosphatase inhibitor cocktail (APE×BIO, K1007) at a 1:5 ratio. The mixture was incubated on ice for 30 min with brief vortexing every 10 min. The lysate was then centrifuged at 14,000 × g for 20 min at 4 °C to remove insoluble components, and the resulting supernatant containing exosomal proteins was carefully collected. The total protein concentration was precisely determined using a BCA Protein Assay Kit (Beyotime, China) according to the manufacturer's instructions. The harvested exosomal protein supernatant samples were mixed with 5× SDS-PAGE loading buffer to reach a 1× final concentration. The mixtures were then denatured at 100 °C for 8 min, or stored at -80 °C for subsequent western blotting (WB) analysis.

**Exosomes identification**

Isolated exosomes were resuspended in 100 µL of 1× PBS and then validated through morphology, particle size, and protein markers. For Transmission electron microscopy (TEM), exosomes were adsorbed onto formvar-carbon-coated copper grids, negatively stained with 2% phosphotungstic acid, and visualized using a JEM-1400 Flash electron microscope (JEOL, Japan) to confirm their typical cup-shaped morphology. Nanoparticle tracking analysis (NTA, NanoSight NS300, Malvern Instruments, UK) was used for the size distribution and concentration measurements of exosomes in liquid suspension from the properties of both light scattering and Brownian motion. Furthermore, the characterization of exosomes was confirmed by measuring the expression of exosome-specific markers TSG101 (ZEN BIO, 381538, 1:1000), the endosomal sorting complexes required for transport (ESCRT) component Alix (Immunoway, YM8063, 1:1000), and EV-associated protein markers HSP70 (ABclonal, A12948, 1:1000) by WB analysis. All assays were performed in triplicate to ensure reproducibility.

**Exosomes labeling and uptake into recipient cells**

To monitor exosomal trafficking, purified exosomes were labeled with the green fluorescent dye PKH67 (Unibio, Shanghai, China). Briefly, exosomes were resuspended in 100 µL of 1× PBS and mixed with PKH67 dye (pre-diluted in Diluent C at a 1:9 ratio). After incubation for 10 min at room temperature in the dark, the labeling reaction was terminated by adding 3% exosome-depleted BSA. To ensure the complete recovery of labeled exosomes and removal of excess dye, 320 µL of exosome isolation reagent (System Biosciences, USA) was added to the mixture, followed by incubation at 4 °C overnight. Then washed with 1× PBS and centrifuged at 110,000 × g for 1 h at 4 °C to collect the exosomes, which were resuspended in 100 µL of complete medium. Recipient cells were then incubated with the PKH67-labeled exosomes for 4 h at 37 °C. After incubation, the cells were washed three times with 1× PBS to remove unbound exosomes, fixed with 4% paraformaldehyde, and the nuclear DNA was stained with DAPI (1:1000) for 1 min. The images were then taken with a fluorescence microscope. The results were analyzed by observing the fluorescence in the cells.

**Identification of potential small open reading frames (ORF)**

We employed the online ORFfinder server (https://www.ncbi.nlm.nih.gov/orffinder/), Pfam server (http://pfam.xfam.org/), and CPC (https://cpc.gao-lab.org/) to assess the coding potential of the long noncoding RNA.

**RNA isolation and qRT-PCR**

Total RNA was isolated using Trizol reagent (Vazyme, Nanjing, China). Complementary DNA (cDNA) was synthesized using the [TransScript Uni](https://www.transgen.com/rt_pcr/879.html) [All-in-One First-Strand cDNA Synthesis SuperMix for qPCR (One-Step gDNA Removal)](https://www.transgen.com/rt_pcr/875.html) [(](https://www.transgen.com/rt_pcr/879.html)[TransGen Biotech](https://www.so.com/link?m=zOm8EPGhcWcqOSOEx9/4CBFe4uLKeOifMnWU0re+p0T/8EI+MYHetPVioGAGDqIaPyHmE/w3UvIDcOlVNDvhQpj0lOgNKdZJCIMjZQXwFt7R0CxDEdGHFjtxP5egRiRuKvSw6HOTdSmKpKpcWP//aHy14JdQVnAf9x1xZMxL0I4Zl1veWTnyu4aotIY4=)[, AU341-02-V2).](https://www.transgen.com/rt_pcr/879.html) qRT-PCR was performed by the Applied Biosystems 7500 Real-Time PCR System and the ChamQ Universal SYBR qPCR Master Mix (Vazyme, Q711-02) with the following modifications: 95 °C for 30 s, followed by 40 cycles, each consisting of denaturation at 95 °C for 10s and annealing extension at 60 °C for 30 s. We chose glyceraldehyde-3-phosphate dehydrogenase (GAPDH) to normalize the target genes' expression levels. Relative RNA abundances were calculated by the standard 2^−ΔΔCt^ method. All of the sequences of primers are shown in **Table S1** and **Table S2**.

**Transwell assay and wound healing assay**

Cell migration was measured by Transwell assays using the 24-well Transwell chambers with 8 µm polycarbonate membranes (Corning Incorporated, USA). A total of 2× 10^5^ cells in 200 µL serum-free medium were added to the upper chamber. The lower chamber contained 800 µL medium with 20% FBS as a chemoattractant. After incubation for 24 h, the cells on the lower surface were fixed with 4% [paraformaldehyde](C:/Users/Lenovo/AppData/Local/youdao/dict/Application/8.9.3.0/resultui/html/index.html#/javascript:;) for 30 min and stained with crystal violet for 20 min. The relative cell number was calculated using a light microscope (Olympus, Tokyo, Japan). For the wound healing assay, cells were inoculated in 6-well or 12-well plates. Subsequently, an artificial linear wound was generated by scratching the monolayer of cells with a sterile 10 µL pipette tip, when the cells reached a density of 90% confluence, and was then added with medium in 1% FBS. Observe the cells under an inverted microscope and monitor the wound closure. The formula to calculate the cell migration rate was as follows: (W0h-W×h) ×100%/W0h, where W0h represents the mean wound width at 0 h, and W×h represents the mean wound width at 24 h, 36 h, 48 h, and 72 h.

**Cell adhesion assay**

Fibronectin (FN) was diluted with RPMI-1640 (BI) to a working concentration of 10 µg/mL, inoculated in 48-well plates, and coated overnight at 4 ℃. The next day, it was placed at 37 ℃ for 1 h, the excess FN solution was absorbed, 5× 10^4^ corresponding cells were inoculated per well, and incubated at 37 ℃ for 1 h. Non-adherent cells were removed by gentle washing with 1× PBS, and the remaining adherent cells were fixed with 4% paraformaldehyde for 1 h, followed by staining with 0.1% crystal violet for 30 min. After three gentle washes with 1× PBS, the adherent cells were imaged and counted using an Olympus optical microscope. The adsorption rate was determined as follows: After the imaging count, the 48-well plate was air-dried, crystal violet was dissolved with 200 µL acetic acid per well for 5 min/ time, and then 100 µL per well was transferred to the 96-well plate, and the OD570 value was measured by an enzyme spectrometer.

**Immunofluorescence (IF) assay**

Cells were transfected with YG-6, 5U-YG-6, or 5U-YG-6mut fused GFP vector, and GFP fluorescence was directly visualized. Cells were transfected with the indicated vectors or siRNAs for 24 h and then seeded the cells in the 96-well plate. These cells were fixed with 4% paraformaldehyde, permeabilized with 0.1% Triton X-100, blocked with 1% BSA, and incubated with the indicated primary antibodies at 4 ℃ overnight. After three washes with 1× PBS, cells were incubated with Alexa Fluor 488 or 594-conjugated secondary antibodies (Invitrogen, 1:500) for 1 h at room temperature in the dark. DAPI was used to stain cellular nuclei for 1 min. The immunofluorescence was observed by laser scanning confocal microscopy. For each channel, all images were acquired with the same settings.

**Immunohistochemistry (IHC) analysis**

Protein expression of paraffin-embedded tissue samples was characterized using IHC staining. Briefly, sections were deparaffinized at 65 °C for 4 h, followed by immersion in xylene and rehydration through a graded ethanol series (100%, 95%, 80%, and 70%). Endogenous peroxidase activity was quenched with a peroxidase blocking reagent for 20 min at 37 °C. For antigen retrieval, sections were heated in citrate buffer (pH 6.0) using a pressure cooker (1600W for 1 min, then 1900W for 2 min), maintained for 10 min, and naturally cooled to room temperature. After blocking with normal goat serum for 30 min at 37 °C, sections were incubated with the indicated primary antibodies at 4 °C overnight (16 h). The following day, the tissue slices were re-warmed at room temperature for 30 min to restore their reactivity. Subsequently, the slices were incubated with Reagent 2 (Amplification Enhancer) for 20 min at 37 °C, followed by Reagent 3 (Enzyme-labeled Polymer) for an additional 20 min at 37 °C. After each incubation step, the sections were thoroughly washed with 1× PBST to ensure the specificity of the immunoreaction. Immunoreactivity was visualized using a DAB chromogen kit, and sections were counterstained with hematoxylin, dehydrated, and mounted. A comprehensive IHC score was calculated as the product of the staining intensity score (0, negative; 1, weak; 2, moderate; 3, strong) and the percentage of positive cells (1, 0%-25%; 2, 26%-50%; 3, 51%-75%; 4, > 75%).

**CCK-8, colony formation, and cell cycle assays**

Cell viability was assessed using a CCK-8 assay (MeilunBio). Briefly, cells (2× 10^3^ cells/well) were seeded into 96-well plates and incubated for 0, 12, 24, 48, and 72 h. At each time point, 10 μL of CCK-8 reagent was added to each well and incubated at 37 °C for 2 h, after which the absorbance was measured at 450 nm using a microplate reader. For the colony formation assay, cells (100 cells/well) were inoculated into 12-well plates and cultured for 10-14 days. The resulting colonies were fixed with 4% paraformaldehyde and stained with 0.1% crystal violet for 30 min. Colonies containing more than 50 cells were counted and photographed. For cell cycle analysis, cells were harvested, fixed in 70% ice-cold ethanol at -20 °C overnight, and subsequently stained with Propidium Iodide (PI) solution (BD Biosciences) for 30 min in the dark. The cells were examined on a FACS Calibur system (BD Biosciences, USA) and the results were analyzed by Flowjo.

**Silver staining assay**

To visualize the protein bands following SDS-PAGE, silver staining was performed using a Silver Staining Kit (CWBIO, CW20125) according to the manufacturer's instructions with minor modifications. Briefly, the gels were rinsed twice with deionized water and fixed in a solution containing deionized water, ethanol, and glacial acetic acid (6:3:1) twice for 15 min each at room temperature. After washing with 10% ethanol and deionized water, the gels were incubated in a sensitizer working solution for 1 min, followed by three quick rinses with deionized water (20 s each). Subsequently, the gels were immersed in a silver nitrate solution and incubated with gentle shaking for 30 min. After washing away excess silver ions, the protein bands were visualized in a developer solution for 2-5 min until the target bands became distinct and clear. The reaction was promptly terminated using a 5% glacial acetic acid stop solution for 5-10 min. The stained gels were then washed three times with deionized water and imaged against a white background to record the differential protein expression profiles.

**Lentiviral production and generation of stable cell lines**

Lentiviral vectors (PCDH-CMV-MCS-EF1α-GFP-Puro) harboring the cDNA sequence (LINC01123, YG-6, 5U-YG-6, and 5U-YG-6mut) were co-transfected into HEK-293FT cells along with the packaging plasmids psPAX2 and pMD2.G at a ratio of 3:2:1 using jetPRIME. After 15 min of incubation at room temperature, the mixture was added dropwise to HEK-293FT cells at 70% confluence. The medium was replaced with fresh DMEM containing 10% FBS after 6 h of incubation. At 48 h post-transfection, the lentivirus supernatant was harvested and filtered through 0.45 µm filter to remove the cell debris, then stored in aliquots at -80 ℃ until use. The target cells were seeded into the 6-well plate at a density of 70% and incubated with the lentiviral supernatant supplemented with 8 µg/mL polybrene to enhance infection efficiency. After 48 h of infection, the virus-containing medium was replaced with fresh complete medium, and the cells were cultured for an additional 24 h to recover their physiological state. Subsequently, stable transformants were selected using 1 µg/mL puromycin. The selection process was maintained for 7 days, during which the puromycin-containing medium was refreshed every 2 days until 100% GFP fluorescence was observed in the experimental groups and total cell death was confirmed in the non-transduced control groups. The final stable cell lines were maintained in medium containing a low concentration of puromycin to ensure sustained transgene expression, and the transduction efficiency was further validated by WB analysis.

**Co-immunoprecipitation (Co-IP) and mass spectrometry (MS) analysis**

For Co-IP assays, cells were lysed in chilled Western and IP cell lysates (P0013, Beyotime) supplemented with a 1% protease and phosphatase inhibitor cocktail. After incubating on ice for 30 min, centrifuge at 4 ℃ for 20 min to remove sediment. To validate exogenous interactions, the supernatants were incubated with anti-GFP (1:100) or anti-Flag (1:100) antibodies overnight at 4 °C. For endogenous Co-IP, the protein extracts were incubated with an anti-YG-6 (1:50) antibody to pull down the endogenous ACTC1 complex, with IgG used as a negative control. The immunocomplexes were subsequently captured overnight at 4 ℃ using 30 µL of pre-washed Protein A/G magnetic beads (Santa Cruz Biotechnology). The beads were washed five times with ice-cold lysis buffer to eliminate non-specific binding. For protein identification, the protein-bound magnetic beads were washed with 1× PBS and submitted directly for MS analysis. For WB validation, the complexes were eluted from the beads by boiling in 1× SDS loading buffer at 100 ℃ for 8 min and separated by SDS-PAGE.

**Molecular docking and domain-specific mutation validation**

To predict the binding mode and critical residues involved in the interaction between YG-6 and ACTC1, rigid protein-protein docking was performed using the GRAMM platform. The three-dimensional structures of target proteins were retrieved from the UniProtKB database, and the optimal binding pose was selected from the top 10 generated conformations based on the lowest binding energy. The binding affinity was further evaluated using PDBePISA to calculate the solvation free energy (ΔG^diss^), and the interaction interface, characterized by hydrogen bonds and hydrophobic contacts, was visualized via PyMOL 3.1. A stable binding complex was identified with a calculated free energy of -21.1 kcal/mol, stabilized by key amino acid residues including ALA-31, ALA-35, ASP-213, and LYS-217. Based on these computational insights, deletion mutations were introduced into the predicted binding domains of YG-6 or ACTC1 using a site-directed mutagenesis kit (C241, Vazyme). The sequences of all mutated primers are shown in **Table S1**. The wild-type (WT) and mutated (MUT) plasmids were then co-transfected into HEK-293FT cells. The role of these specific motifs in the protein complex was validated via exogenous Co-IP assays.

**RNA immunoprecipitation (RIP) assay**

The interaction between specific proteins (YG-6 or ACTC1) and the lncRNA LINC01123 was investigated using an RNA Immunoprecipitation (RIP) Kit (Catalog Bes5101; BersinBio, Guangzhou, China) following the manufacturer's instructions. Briefly, approximately 2× 10^7^ cells were harvested and lysed in chilled polysome lysis buffer supplemented with protease and RNase inhibitors. To minimize DNA interference, the lysates were treated with DNase for 10 min at 37 °C, followed by centrifugation to collect the clarified supernatant. A fraction of the supernatant (10%) was reserved as the Input control, while the remainder was divided and incubated with 5 μg of primary antibodies against YG-6, ACTC1, or Rabbit IgG (negative control) overnight at 4 °C with gentle rotation. Due to the absence of previously reported positive controls for YG-6 and ACTC1, an anti-SNRNP70 antibody (CS203216) and U1 snRNA primers (CS203215) were utilized as a validated positive control system to ensure the technical reliability of the assay.

The immunocomplexes were captured using pre-washed Protein A/G magnetic beads for 1 h at 4 °C, followed by a series of stringent washes using polysome washing buffers to eliminate non-specific binding. The protein-bound RNA was eluted via Proteinase K digestion at 55 ℃ and purified using the phenol-chloroform-isoamyl alcohol (125:24:1) extraction method. The purified RNA was then reverse-transcribed into cDNA, and the enrichment of LINC01123 and U1 snRNA (positive control) in the immunoprecipitated fractions was quantified via qRT-PCR and analyzed using the Fold Enrichment method. For each sample (IP groups or IgG control), the average Ct values were first normalized to their respective Input values to obtain the △Ct, and the relative abundance of the target RNA was then calculated as 2^-△Ct^. Finally, the enrichment levels of all IP groups were normalized to the 2^-△Ct^ value of the non-specific IgG group to determine the Fold Enrichment (Relative to IgG).

**Reverse transcription of low-yield RNA**

To synthesize cDNA from low-concentration RNA templates, such as those derived from RNA immunoprecipitation (RIP) or exosomes, the RevertAid First Strand cDNA Synthesis Kit (Thermo Scientific, K16215) was utilized according to the manufacturer's instructions. Initially, to ensure the removal of genomic DNA, RNA preparations were treated with 1 U of RNase-free DNase I in a 10 µL reaction volume containing 10× Reaction Buffer with MgCl_2_ for 5 min at 37 ℃. The reaction was terminated by adding 1 µL of 50 mM EDTA and heating at 65 ℃ for 10 min. Subsequently, first-strand cDNA was synthesized in a 20 µL reaction system. For the priming step, the Random Hexamer Primers (1 µL each) was added to the RNA template. After an initial denaturation at 65 ℃ for 5 min, the reaction was supplemented with 5× Reaction Buffer, RiboLock RNase Inhibitor (20 U), 10 mM dNTP Mix, and RevertAid Reverse Transcriptase (200 U). The mixture was incubated at 25 ℃ for 5 min, followed by 60 min at 42 ℃ to facilitate efficient reverse transcription. Finally, the enzyme was inactivated by heating at 70 ℃ for 5 min. The resulting cDNA was stored at -80 ℃ or directly used for downstream qPCR analysis.**Supplementary Materials**

**
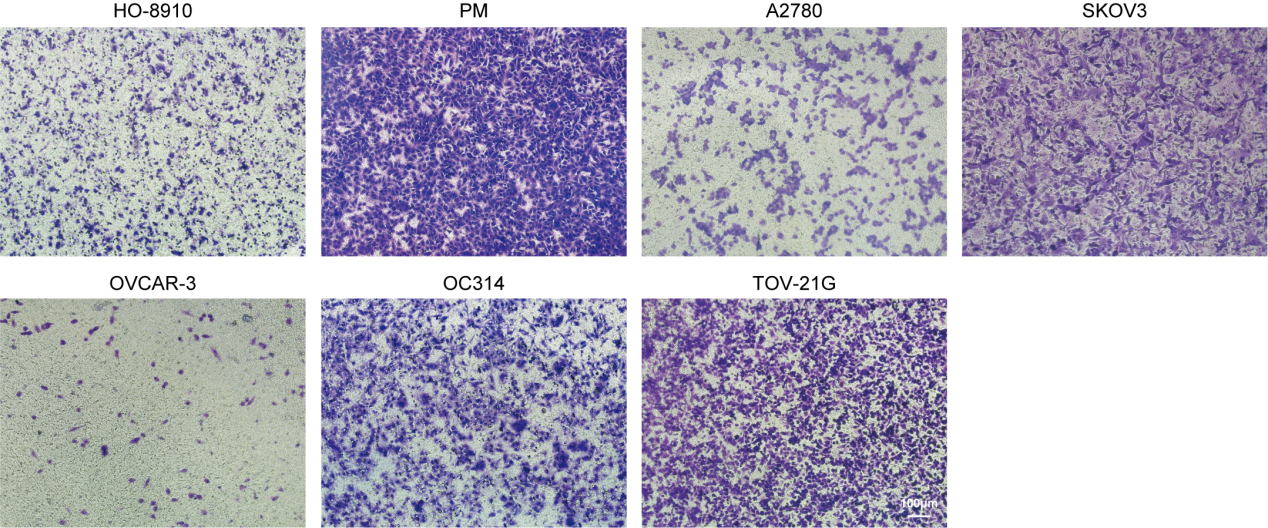
Fig. S1 The migration abilities of various OC cells**

Representative images of cell migration assessed by Transwell assay in HO-8910, PM, A2780, SKOV3, OVCAR-3, OC314, and TOV-21G cell lines. Scale bar: 100 µm.

**
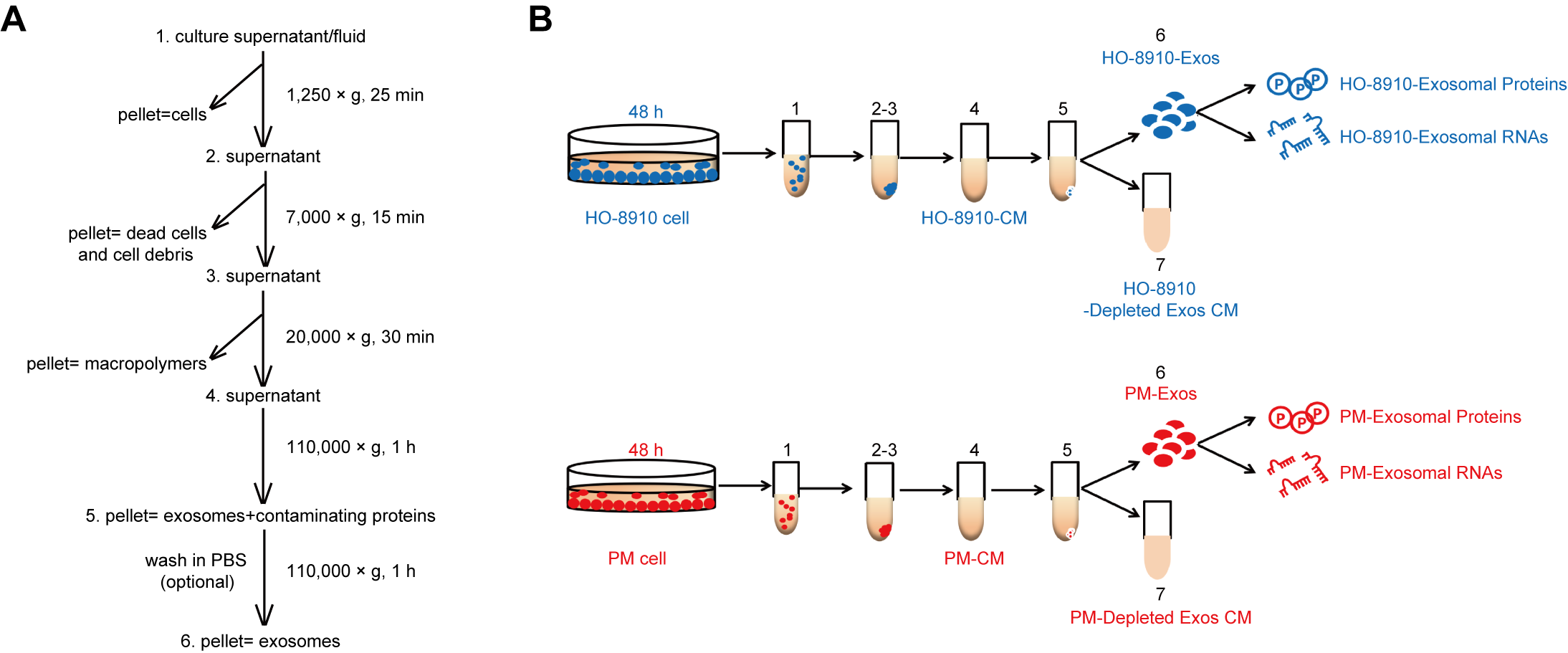
Fig. S2 The process of exosomes ultracentrifugation**

**(A)** Flow chart of ultracentrifugation extraction of exosomes. **(B)** Cartoon diagram of the steps of exosomes, conditioned medium (CM), protein, and RNA extraction. PM cells in red, HO-8910 cells in blue. The numbers 1-7 are marked as described. 1, culture supernatant/fluid; 2-3, cell debris; 4, CM; 5, precipitate and supernatant; 6, Exos; 7, Depleted Exos CM.

**
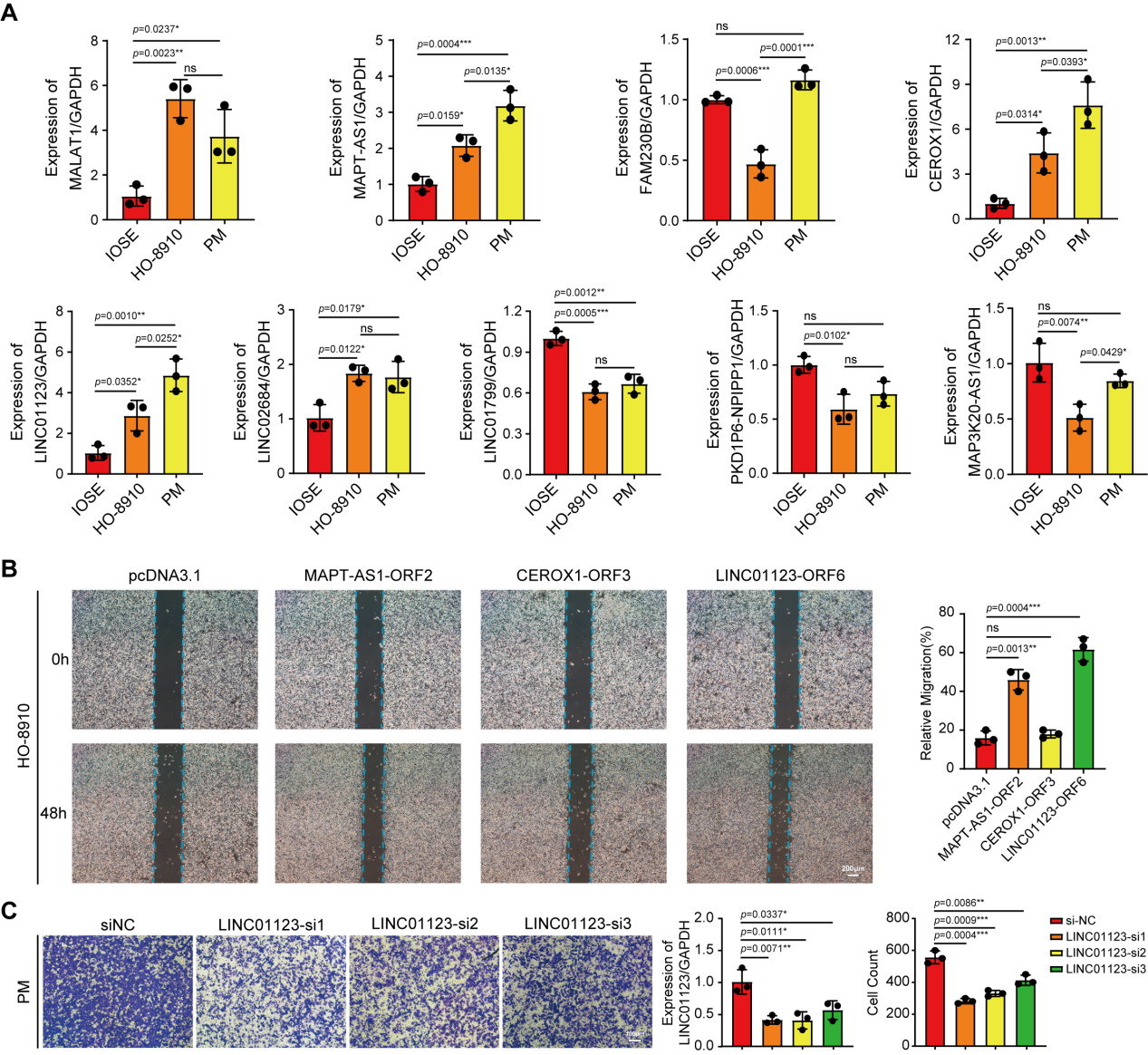
Fig. S3 LINC01123 is highly expressed in HMOs and participates in OC cell migration**

**(A)** qPCR was used to detect 9 candidate lncRNAs' expression in IOSE, HO-8910, and PM cells. **(B)** Wound healing assay was used to detect the migration ability of 3 candidate lncRNA-ORFs with coding potential in OC cells. Scale bar: 200 µm. **(C)** Transwell assay was used to detect the effect of silencing of LINC01123 on OC cell migration abilities. Scale bar: 100 µm. The data are represented as the mean ± SD from three independent experiments (A-C), and the *p* value was determined by one-way analysis of variance (ANOVA) followed by Tukey's (A) or Dunnett's post-hoc test (B-C). All of the experiments were performed in triplicate. **p* < 0.05, ***p* < 0.01, or ****p* < 0.001, ns indicates no significance.

**
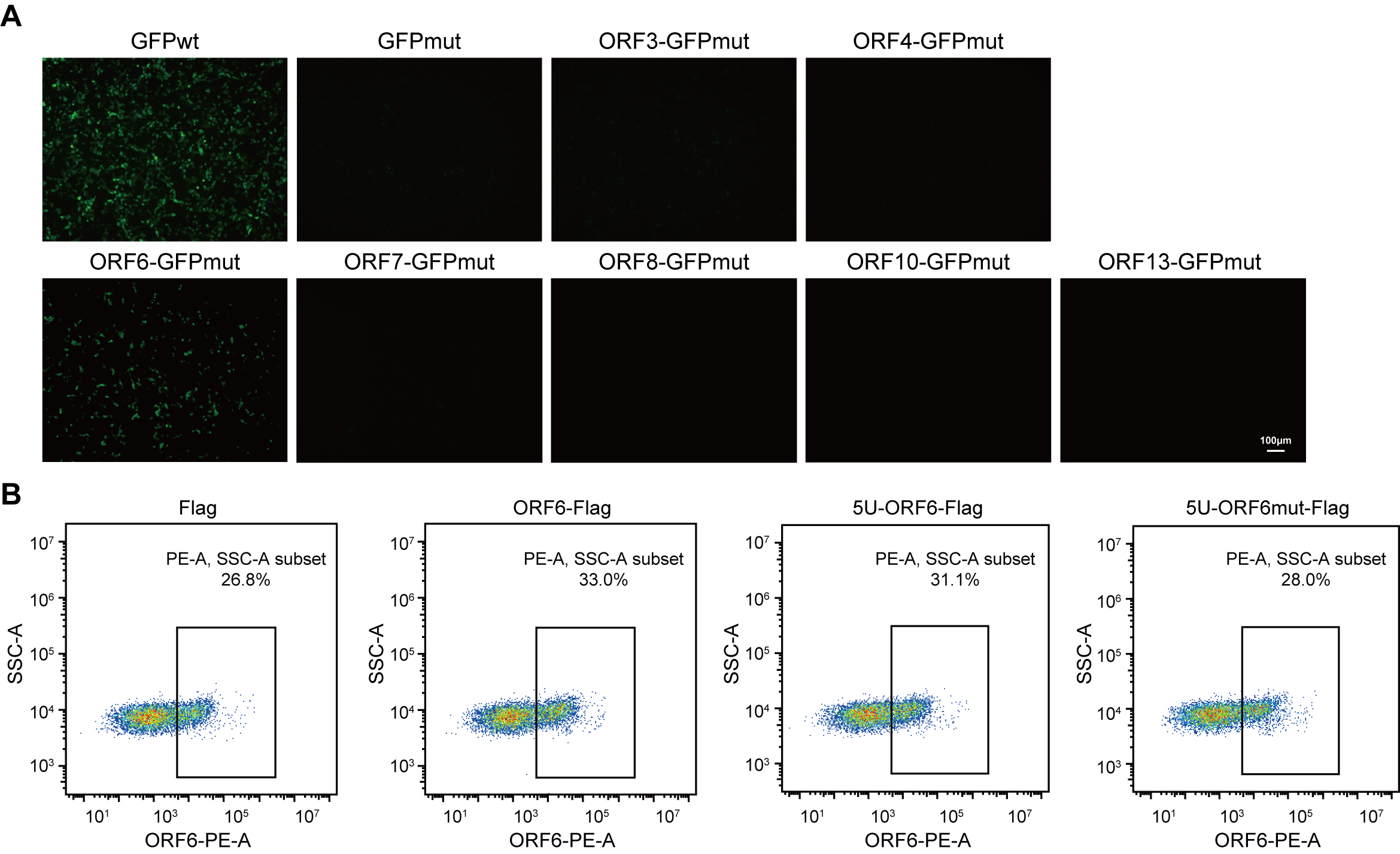
Fig. S4 LINC01123 encodes a micropeptide, ORF6**

**(A)** Representative fluorescence images of ORFs (>30 aa) from the full-length LINC01123 fused to the N-terminus of GFP. Scale bars: 100 µm. **(B)** Detection of ORF6-Flag fusion protein expression by flow cytometry using an ORF6 antibody.

**
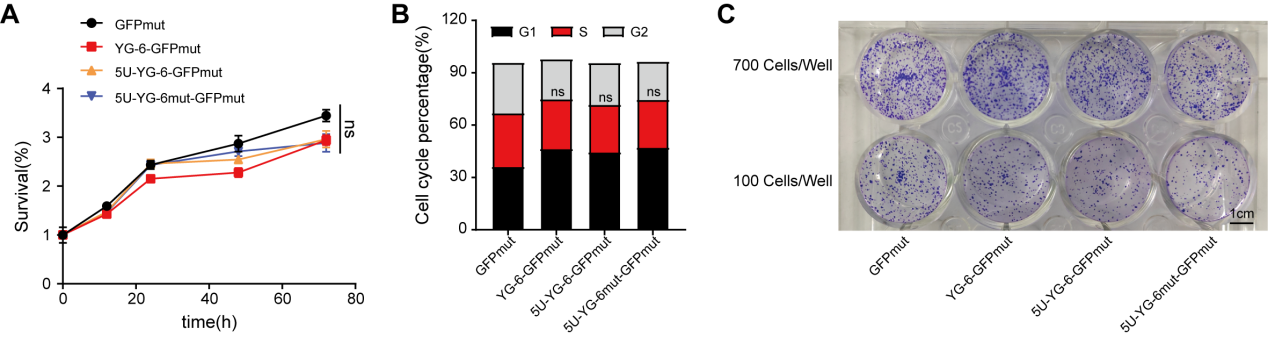
Fig. S5 YG-6 did not affect OC cell proliferation**

**(A)** Cell proliferation was assessed by CCK-8 assay at the indicated time points (0, 12, 24, 48, and 72 h) in HO-8910 cells transfected with YG-6, 5U-YG-6, or 5U-YG-6mut. **(B)** Cell cycle distribution (G1, S, and G2 phases) was analyzed by flow cytometry following transfection with the indicated constructs. **(C)** Representative images of the colony formation assay performed to evaluate proliferative capacity. Scale bar: 1 cm. All experiments were performed in triplicate, and data are presented as the mean ± SD. Statistical significance was determined as follows: For the CCK-8 proliferation curves (A), a two-way ANOVA was employed to compare differences across multiple time points and groups. For cell cycle distribution (B) and colony formation (C), a one-way ANOVA followed by Tukey's post-hoc test was used for multiple group comparisons. ns, not significant (*p* > 0.05).

**
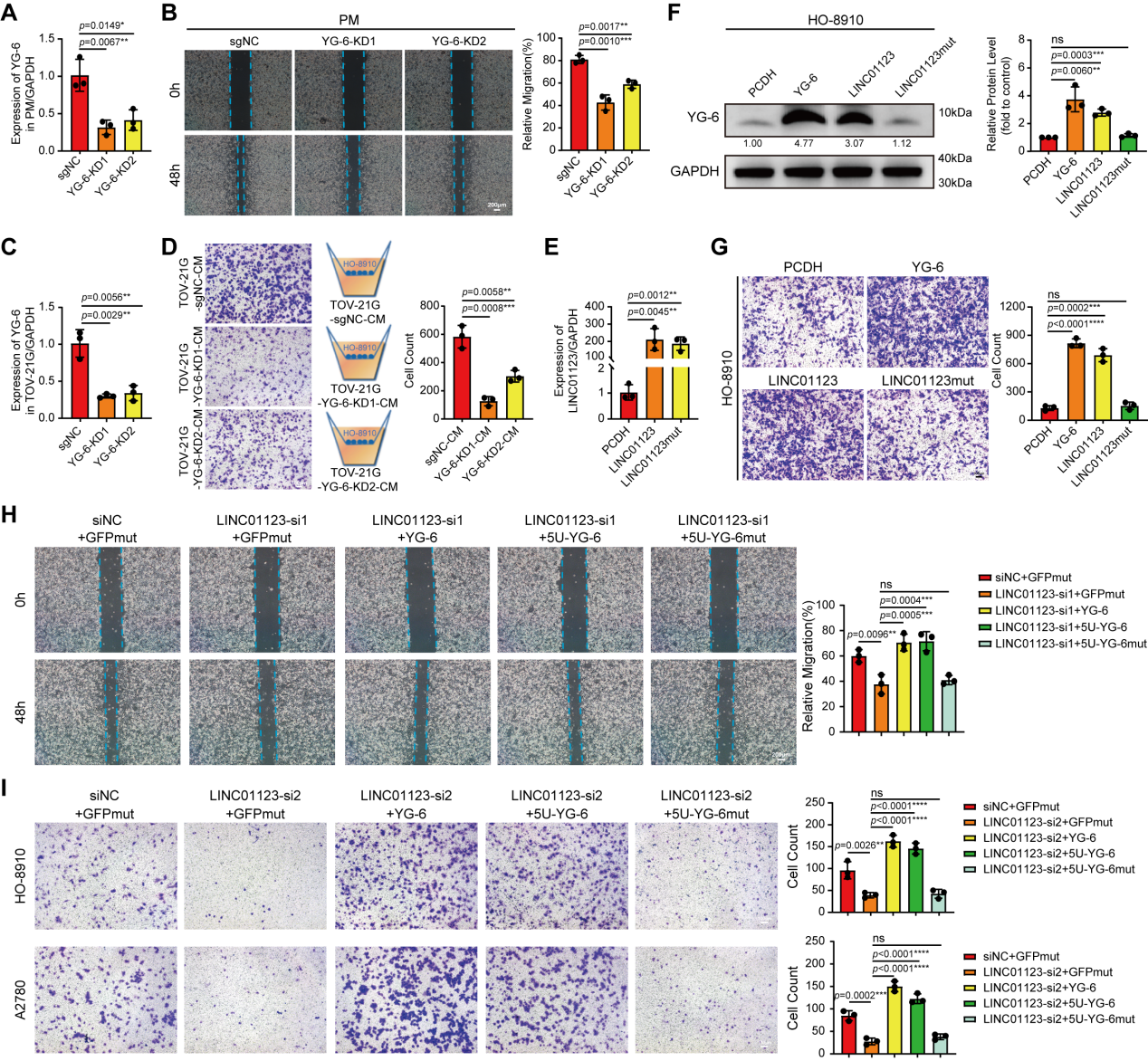
Fig. S6 YG-6, rather than LINC01123 itself, promotes OC cell migration**

**(A)** The knockdown efficiency of YG-6 in PM was detected by qPCR assay. **(B)** Wound healing assay was used to detect the migration ability of YG-6-KD expression in PM. Scale bar: 200 µm. **(C)** Determination of YG-6 knockdown efficiency in TOV-21G cells using qPCR. **(D)** Representative images of cell migration in HO-8910 were co-cultured with TOV-21G^YG-6-KD^. Scale bar: 100 µm (Left). The number of cells was counted by ImageJ (Right). **(E)** qPCR was used to detect the expression of LINC01123 in different treatment groups. **(F)** WB was used to determine the expression of YG-6 in different treatment groups. **(G)** Transwell assay was used to determine the migration rate of the indicated cells. Scale bar: 100 µm. **(H)** The effect of co-transfection with YG-6, 5U-YG-6, and 5U-YG-6mut after silencing LINC01123 on different OC cell migration abilities was detected by wound healing assays. Scale bar: 200 µm. **(I)** The effect of co-transfection with YG-6, 5U-YG-6, and 5U-YG-6mut after silencing LINC01123 on different OC cell migration abilities was detected by Transwell. Scale bar: 100 µm. Each experiment was performed in triplicate. Statistical significance was determined by one-way ANOVA followed by Dunnett's or Tukey's post-hoc test (A-I) for comparisons among multiple groups. The data are represented as the means ± SD. **p* < 0.05, ***p* < 0.01, ****p* < 0.001, or *****p* < 0.0001, ns indicates no significance.

**
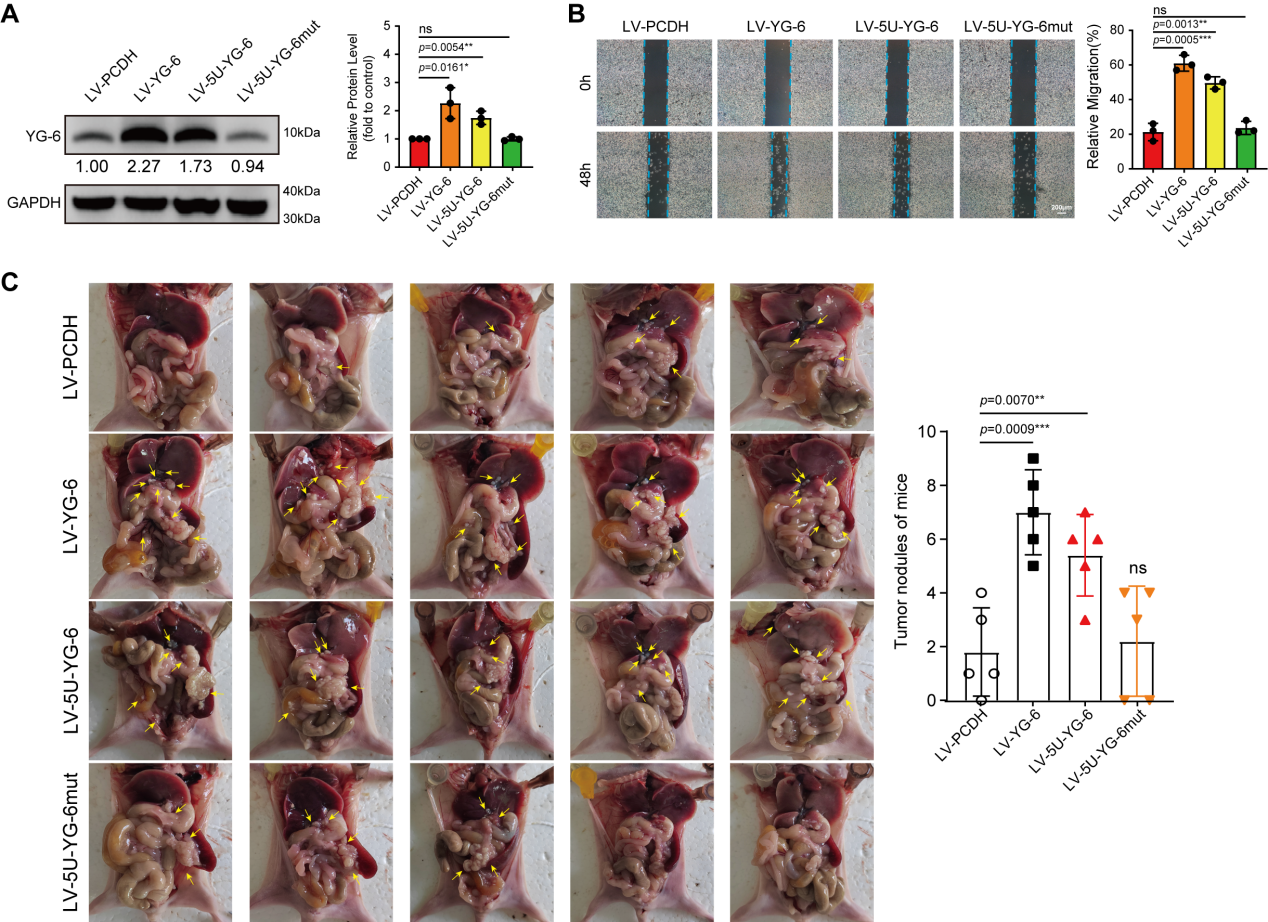
Fig. S7 Verification of YG-6 stable overexpression efficiency and migration function**

**(A)** The efficiency of YG-6 stable overexpression was detected by WB. **(B)** Functional verification of stable YG-6 overexpressed OC cell lines. Scale bar: 200 µm. **(C)** The images and quantitative analysis of metastatic nodules in the abdomens of mice (n=5 mice per group). Each experiment was performed in triplicate (A-B). Statistical significance was determined by one-way ANOVA followed by Dunnett's post-hoc test (A-C) for comparisons among multiple groups. The data are represented as the means ± SD. **p* < 0.05, ***p* < 0.01, or ****p* < 0.001, ns indicates no significance.

**
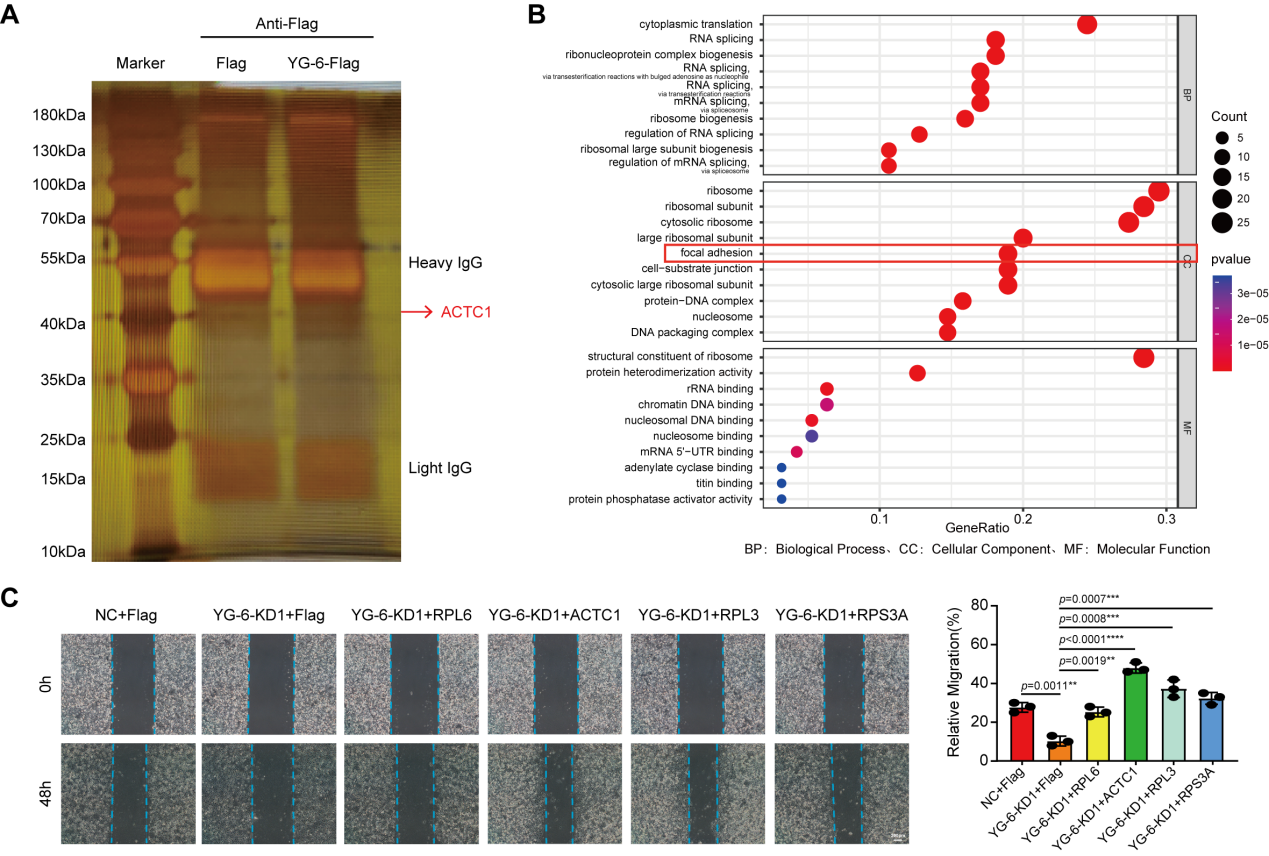
Fig. S8 High expression of ACTC1 was associated with poor prognosis in OC**

**(A)** Proteins that interacted with YG-6 were identified by Co-IP together with MS. **(B)** Proteins that interacted with the YG-6 peptide were shown to participate in focal adhesion. **(C)** Co-transfection of the top 4 genes constructs with YG-6 KD1 into OC cells, and the migration was assessed by the wound healing assay. The data are represented as the mean ± SD from three independent experiments, and the *p* value was determined by one-way ANOVA followed by Tukey's post-hoc test (C). ***p* < 0.01, ****p* < 0.001, or *****p* < 0.0001.

**
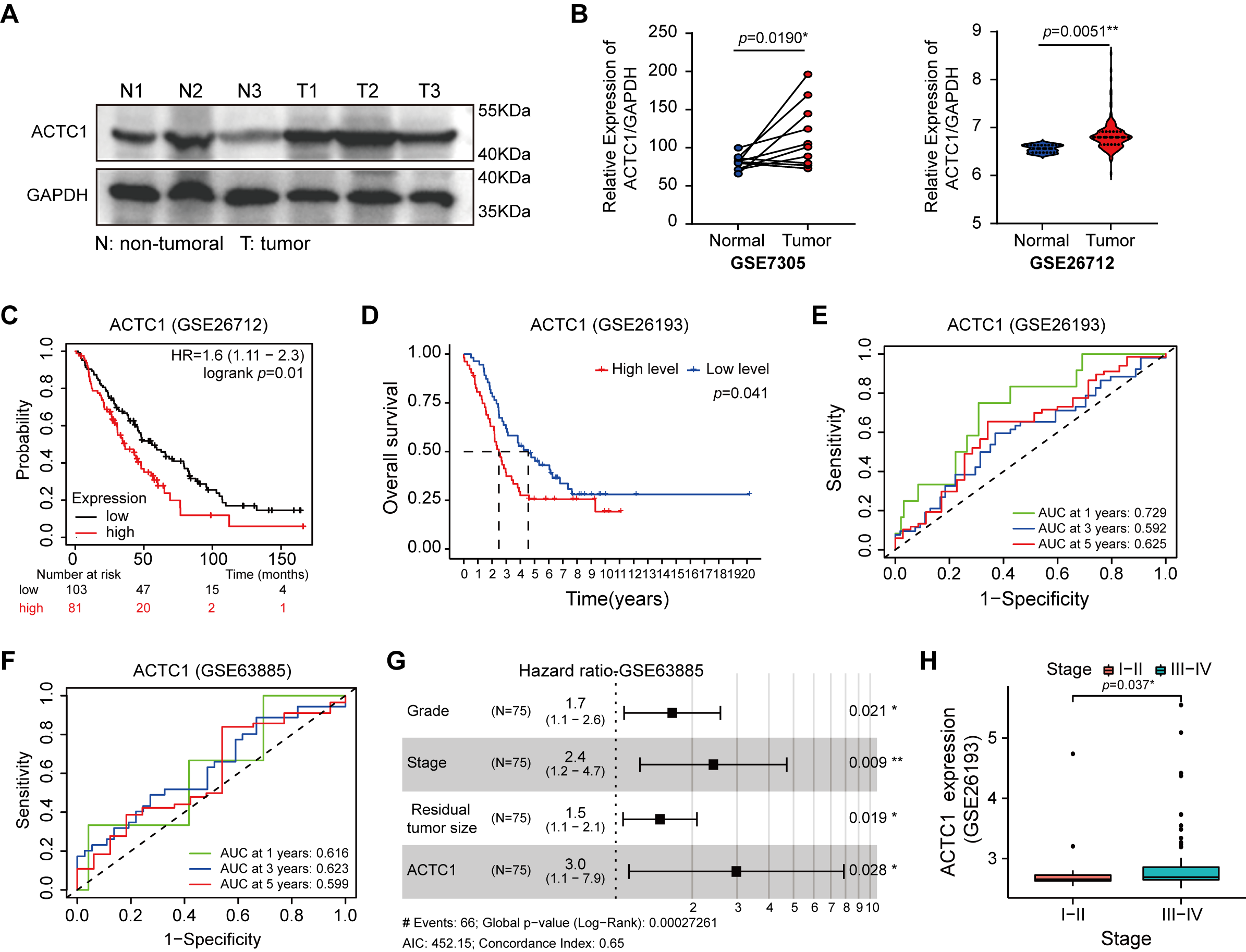
Fig. S9 High expression of ACTC1 was associated with poor prognosis in OC**

**(A)** ACTC1 levels were detected in the OC tissues (T) and the non-tumoral tissues (N). **(B)** Datasets from GSE7305 and GSE26712 indicate ACTC1 expression levels in OC tissues and normal samples. **(C-D)** Kaplan‐Meier survival curves were produced for high and low ACTC1 expression in the OC samples from the GSE26712 datasets and GSE26193 datasets. ACTC1 expression was associated with poor overall survival (OS) in OC patients. **(E-F)** ROC curves displaying the sensitivity and specificity of ACTC1 for the diagnosis of OC patients from the GSE26193 datasets and GSE63885 datasets. **(G)** Meta-analysis depicting forest plots of ACTC1 expression as a univariate predictor of OS. **(H)** GSE26193 datasets showed that ACTC1 was highly expressed in OC patients with high stage.

**
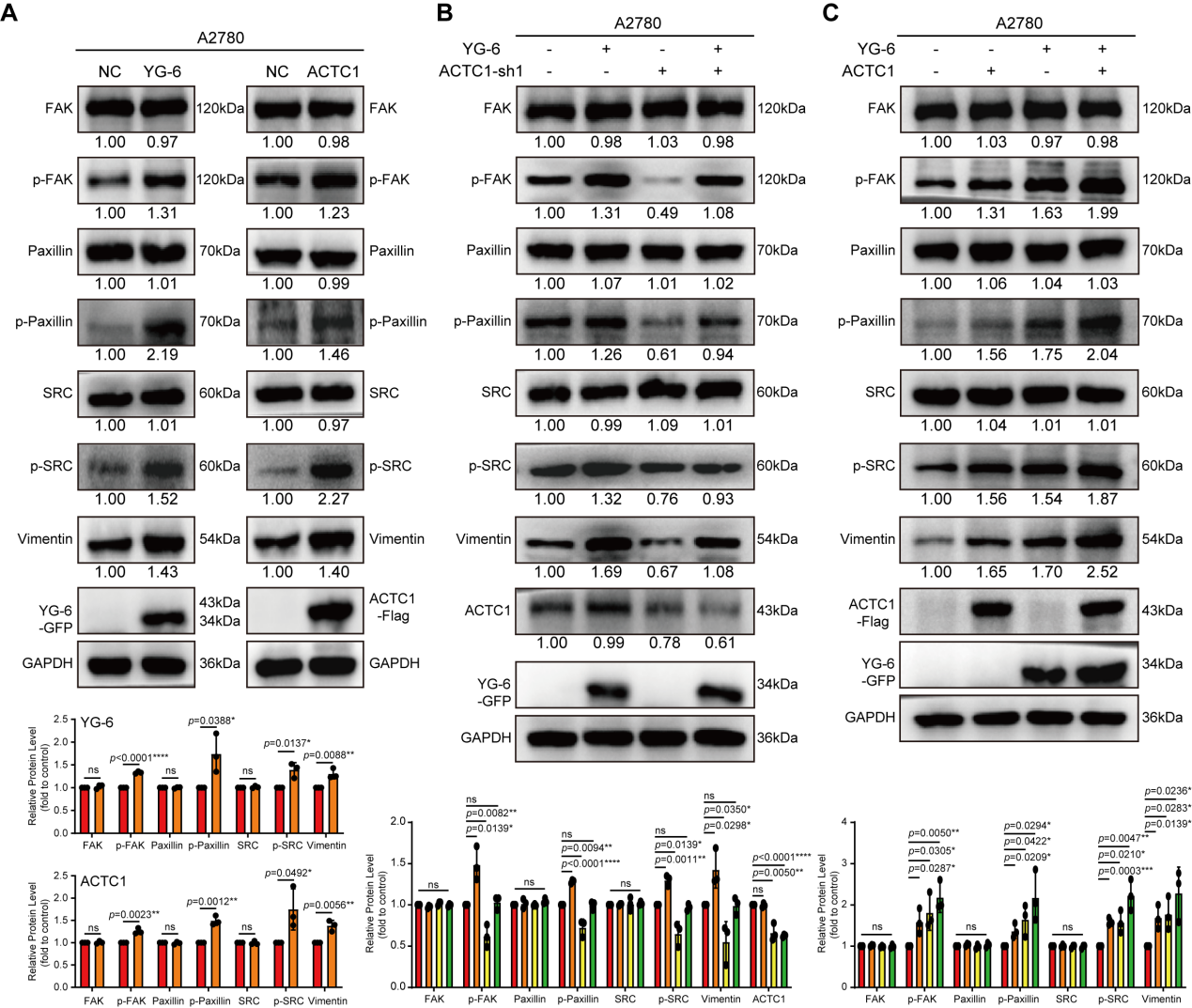
Fig. S10 YG-6 and ACTC1 participate in the focal adhesion signaling pathway**

**(A)** WB analysis of the focal adhesion signaling pathway markers in A2780 cells following over-expression of YG-6 (Left) or ACTC1 (Right). **(B)** WB analysis of signaling pathways in YG-6-overexpressing cells with ACTC1 knockdown. A2780 cells were co-transfected with YG-6 expression vectors and either ACTC1-sh or a negative control shRNA. Protein levels of focal adhesion signaling pathway markers were determined at 48 h post-transfection. **(C)** WB analysis of signaling pathways following co-overexpression of YG-6 and ACTC1. A2780 cells were transfected with YG-6 and ACTC1 expression plasmids individually or in combination. Protein levels of focal adhesion signaling pathway markers were determined at 48 h post-transfection. Quantitative analysis of protein levels (normalized to GAPDH) based on gray value densitometry from three independent experiments (A-C). The data are represented as the means ± SD. Statistical significance was determined using one-way ANOVA followed by Dunnett's or Tukey's post-hoc test (A-G). Each experiment was performed in triplicate. **p* < 0.05, ***p* < 0.01, ****p* < 0.001, or *****p* < 0.0001, ns indicates no significance.

**
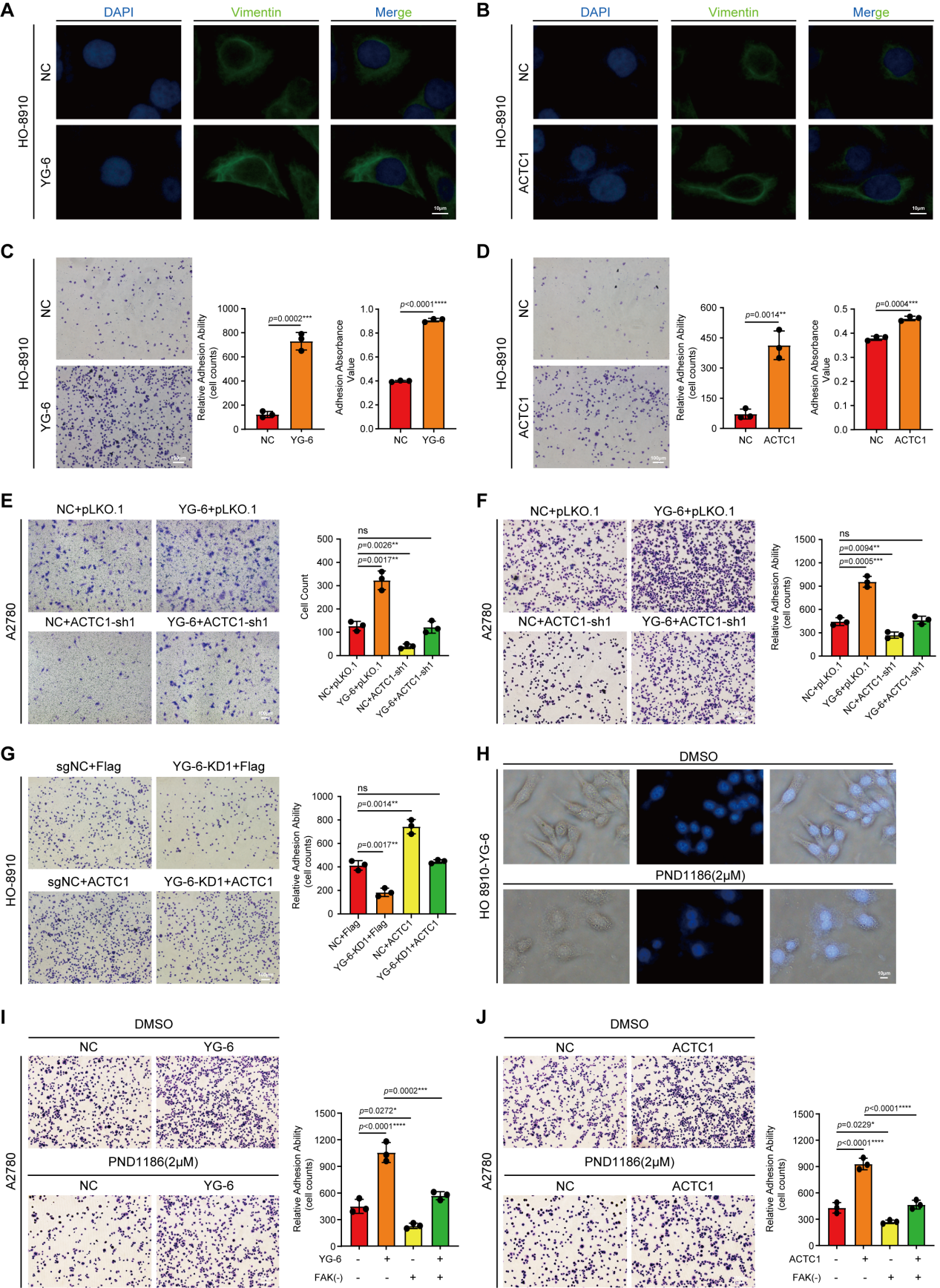
Fig. S11 YG-6 and ACTC1 can promote OC cell adhesion**

**(A, B)** IF staining was performed to detect Vimentin expression in HO-8910 cells following transfection with YG-6 **(A)** or ACTC1 **(B)**. Blue fluorescence indicates DAPI-stained nuclei; green fluorescence indicates Vimentin protein; Merge panels show the overlay of DAPI and Vimentin signals. Scale bar: 10 µm. **(C, D)** Cell adhesion assays were performed to evaluate the effects of YG-6 **(C)** or ACTC1 **(D)** transfection on HO-8910 cells. Scale bar: 100 µm. Quantitative analysis of adhesion ability was performed by cell counting and absorbance measurement. **(E-F)** Co-transfected with YG-6 and either ACTC1-sh1 or a negative control (pLKO.1) in A2780 cells to assess migratory **(E)** and adhesion capacity **(F)**. Scale bar: 100 µm. **(G)** Co-transfection with ACTC1 can restore the inhibitory effects of YG-6 knockdown on cell adhesion in OC cells. Scale bar: 100 µm. **(H)** Representative fluorescence micrographs showing the morphology of HO-8910-YG-6 cells treated with DMSO or the FAK inhibitor PND1186 (2 µM). Scale bar: 10 µm. **(I-J)** A2780 cells were transfected with YG-6 **(I)** or ACTC1 **(J)** and subsequently treated with the FAK inhibitor PND1186 (2 µM) or DMSO control. Cell adhesion capacity was evaluated by counting adherent cells per field. Scale bar: 100 µm. The data are represented as the means ± SD, and the *p* value was determined by two-tailed Student's t test (C-D) or one-way analysis of variance (ANOVA) followed by Dunnett's post-hoc test (E-G) or Tukey's (I-J). Each experiment was performed in triplicate. **p* < 0.05, ***p* < 0.01, ****p* < 0.001, or *****p* < 0.0001, ns indicates no significance.
