# Supplementary figures and images for: Micropeptide YG-6 encoded by exosomal LINC01123 derived from highly migratory ovarian cancer cells promotes tumor progression
Source: Mol Cancer. 2026 Mar 5;25:100. doi: 10.1186/s12943-026-02621-w (PMC13072579; doi:10.1186/s12943-026-02621-w)

**Original images of western blotting**


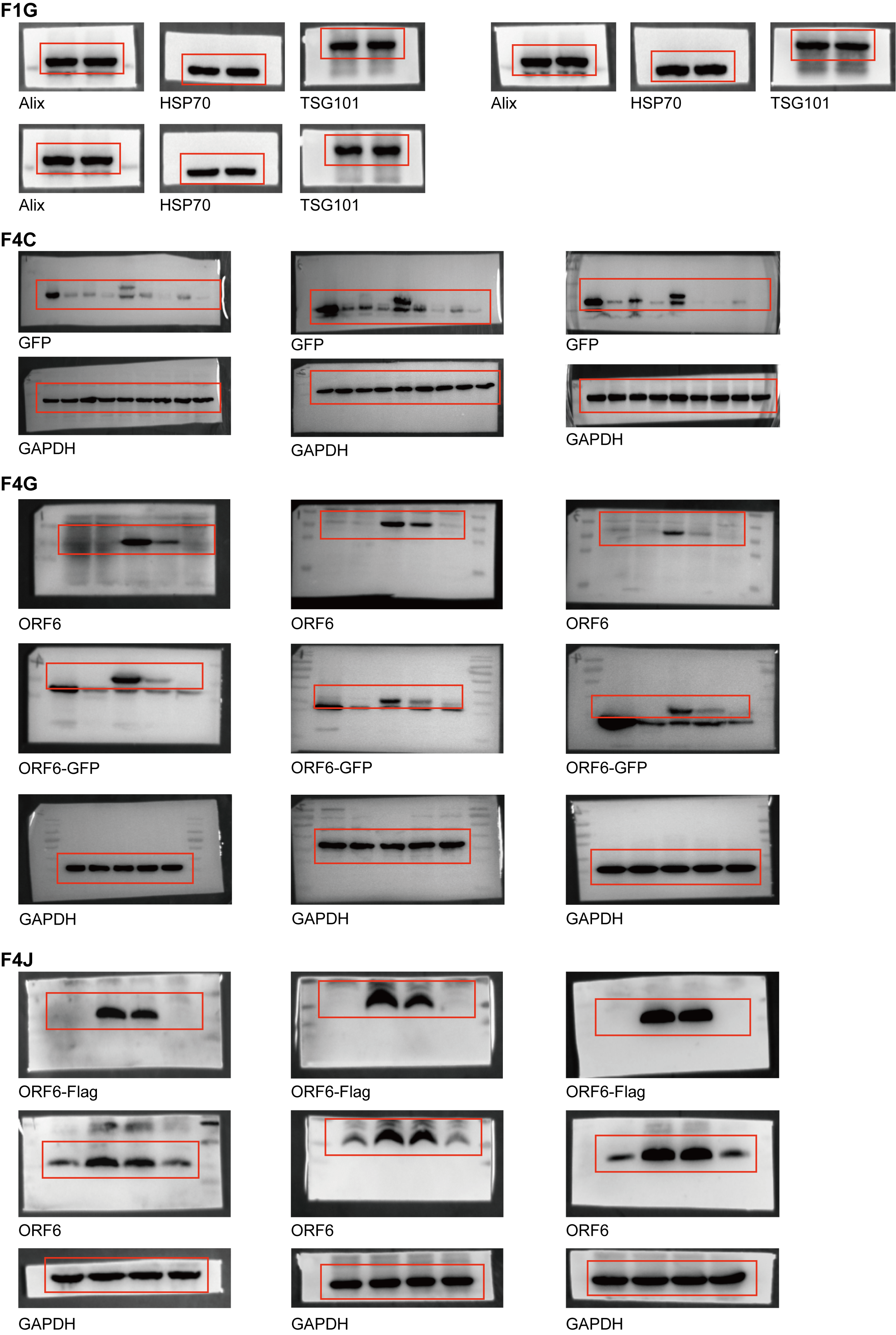


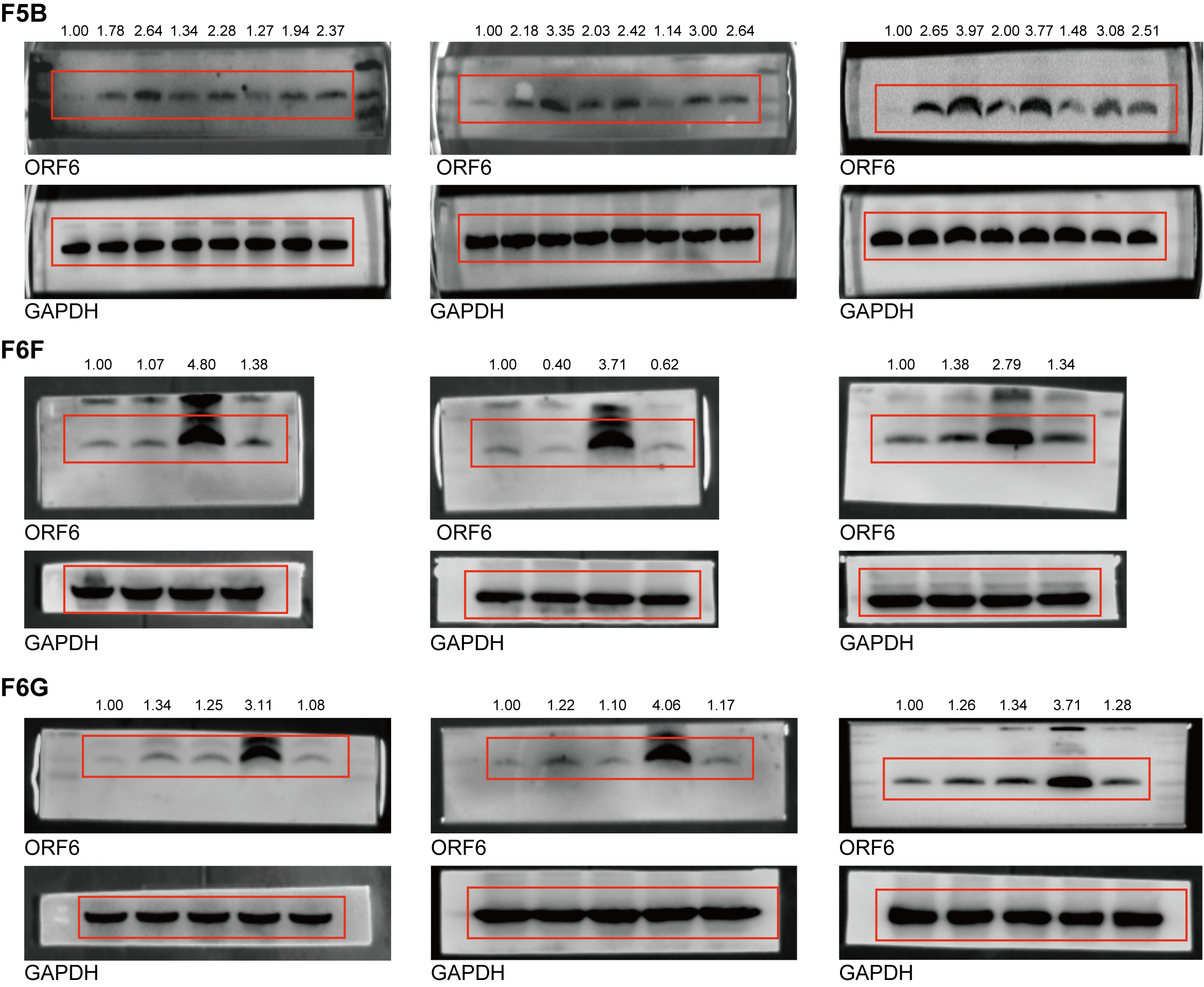


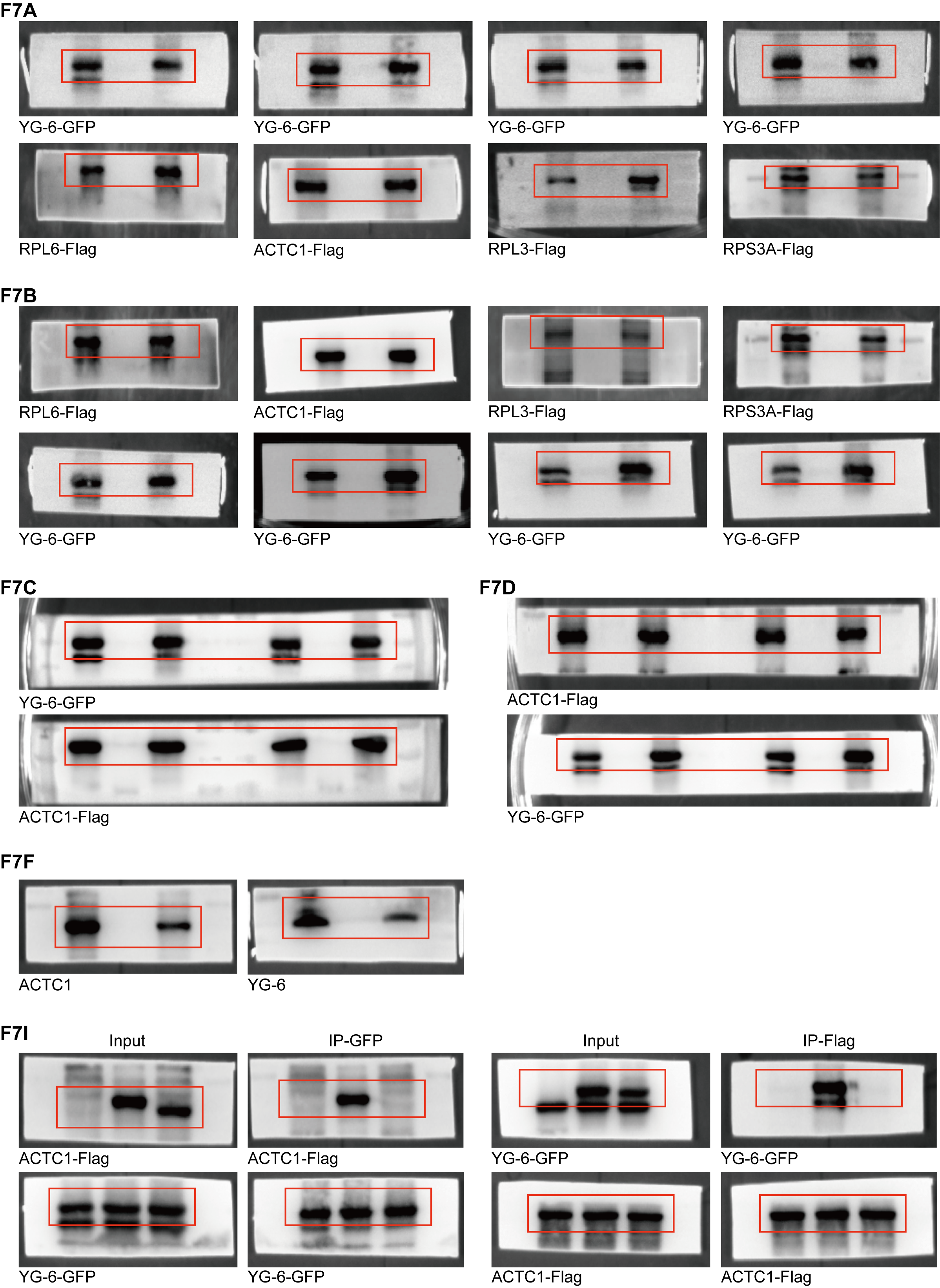


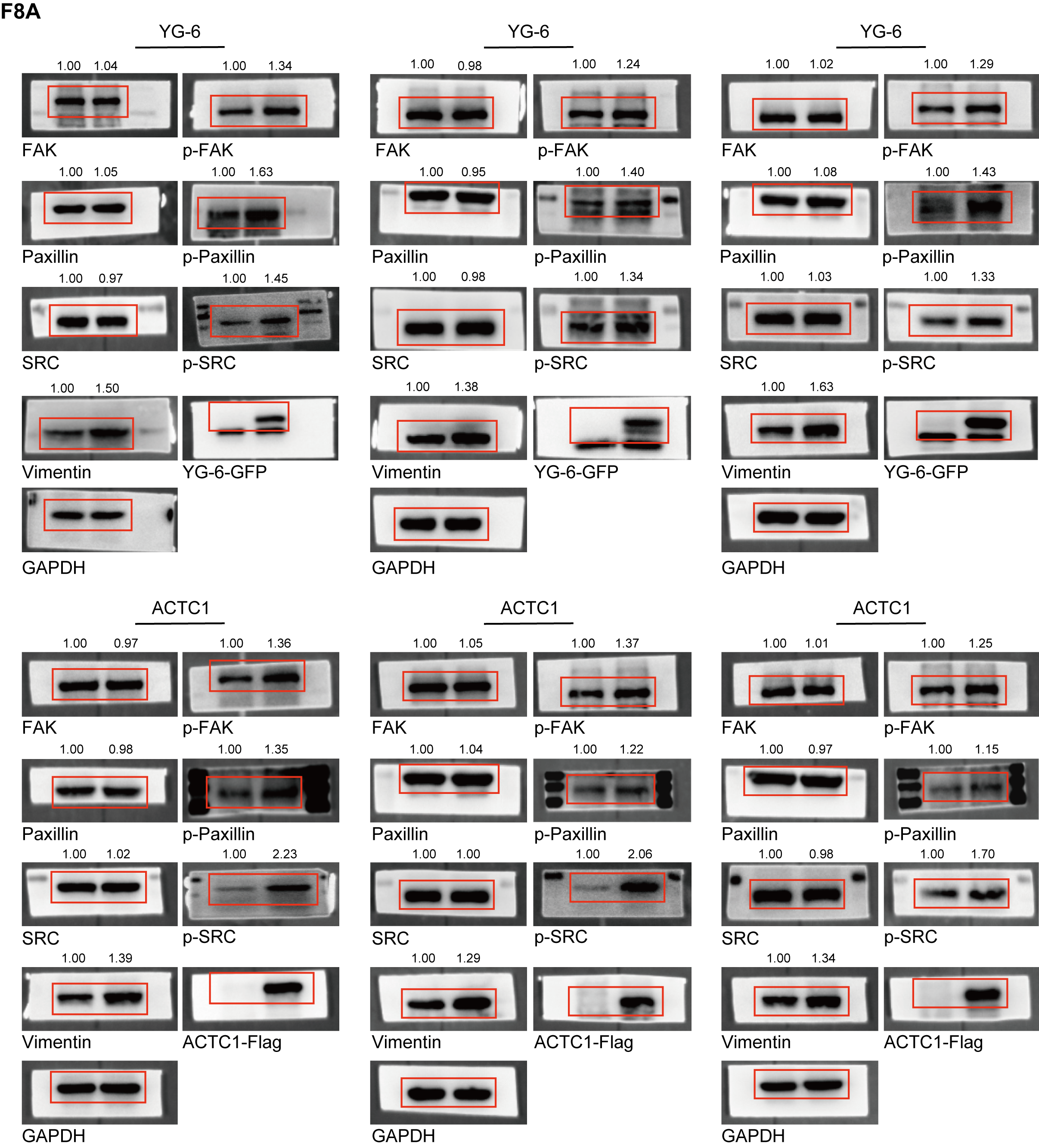


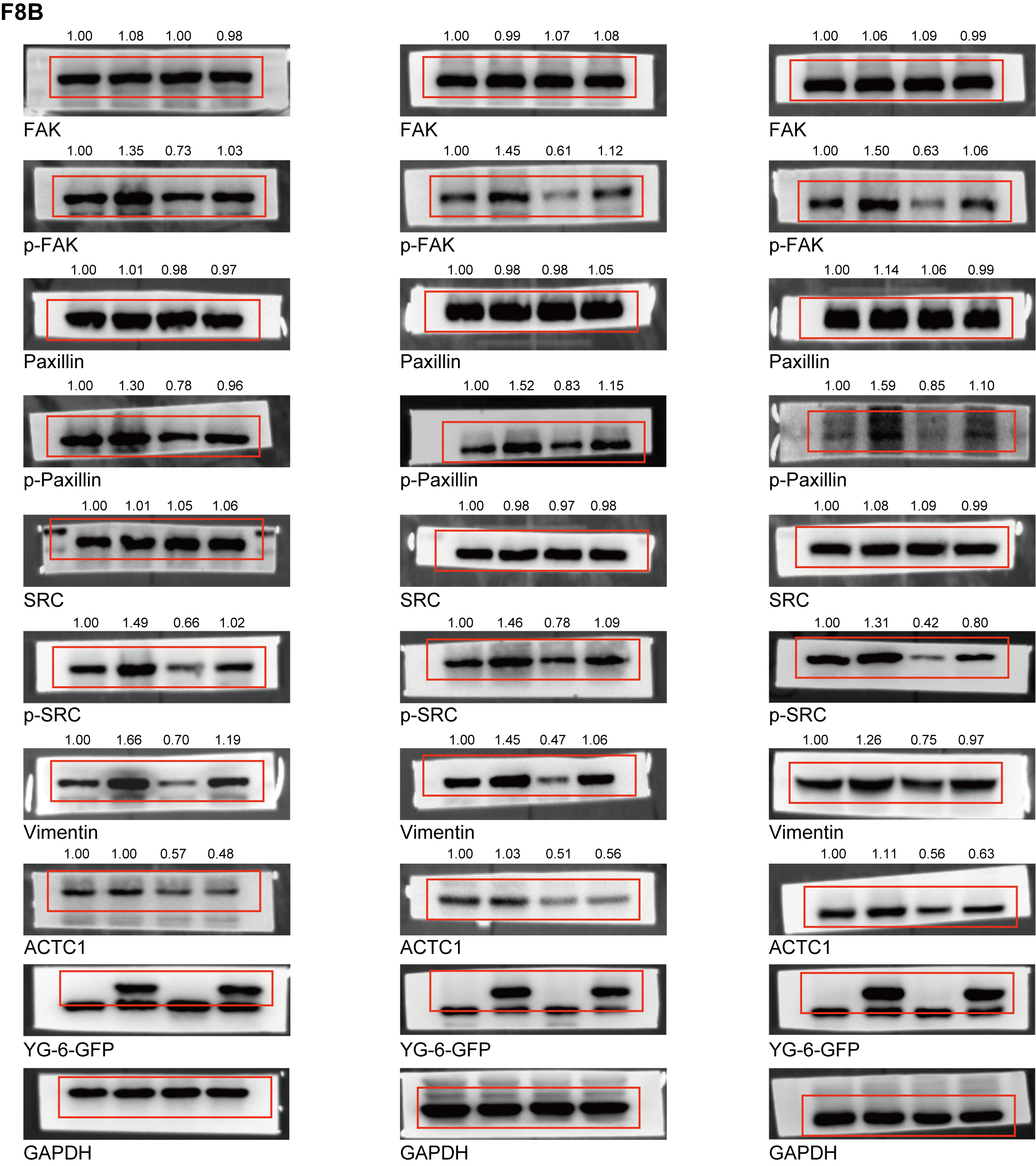


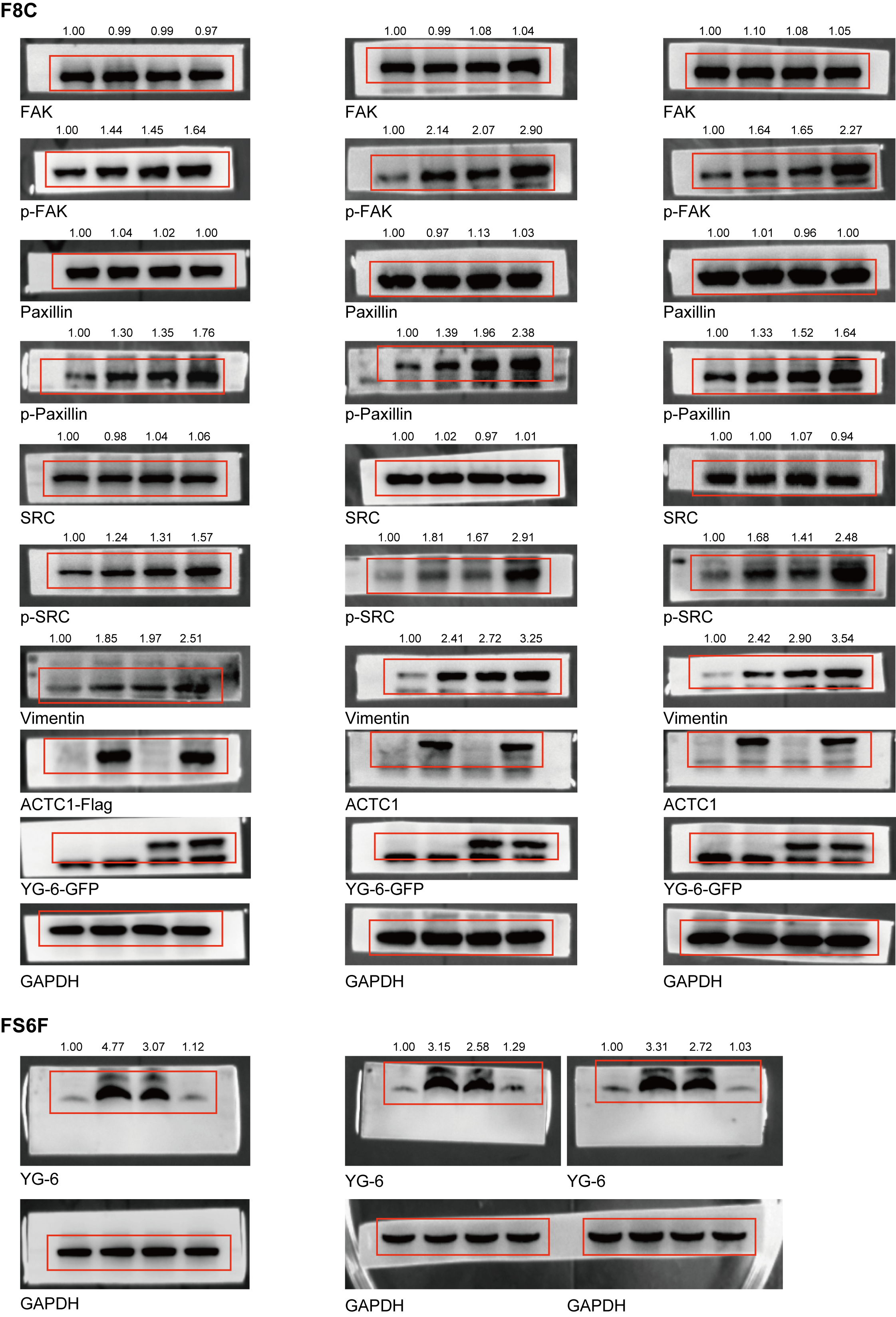


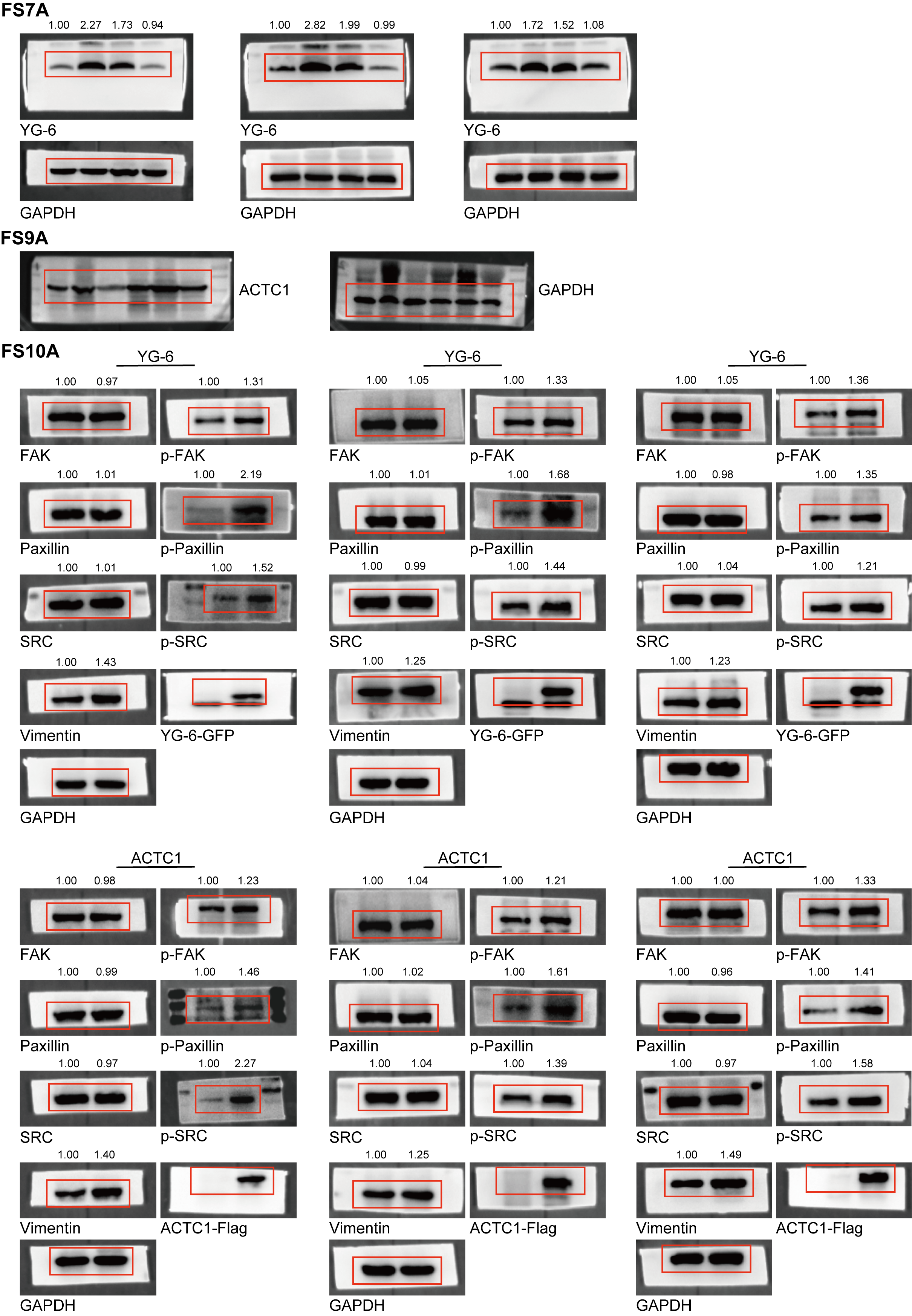


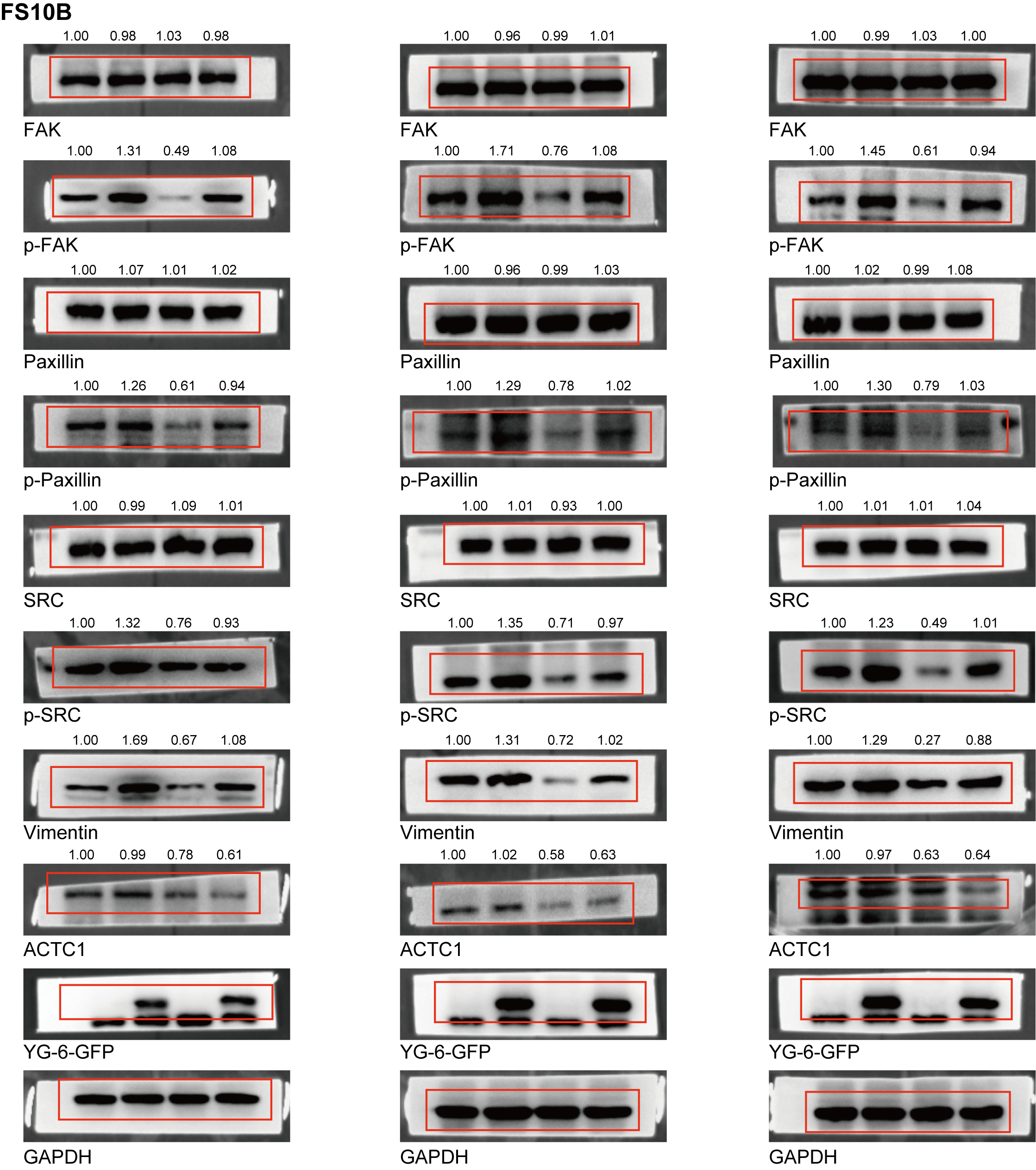


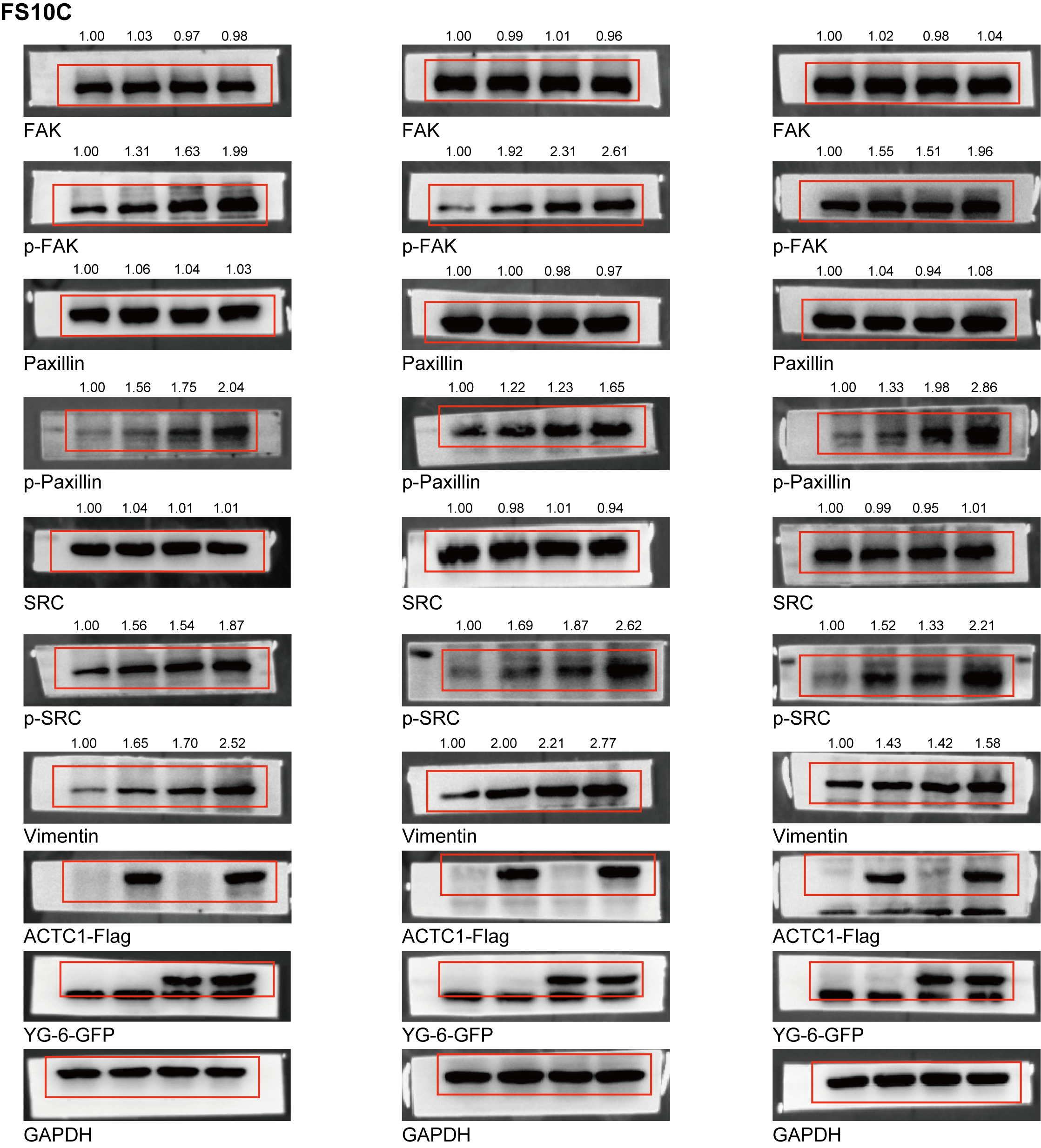

Supplement: Supplementary file 2 — Supplementary Material 2. [file 12943_2026_2621_MOESM2_ESM.doc]
